# Sexual Function, Activity and Distress 24 Months After Surgical Menopause: What Happens After Menopause (WHAM)—A Prospective Controlled Study

**DOI:** 10.1111/1471-0528.70158

**Published:** 2026-01-22

**Authors:** Martha Hickey, Trevor Tejada‐Berges, Susan M. Domchek, Efrosinia O. Krejany, Alison Brand, Peixuan Li, Sabine Braat, Sheryl Kingsberg

**Affiliations:** ^1^ Department of Obstetrics, Gynaecology and Newborn Health University of Melbourne and the Royal Women's Hospital Melbourne Victoria Australia; ^2^ Gynaecological Oncology Service, Chris O'Brien Lifehouse Sydney Australia; ^3^ Basser Center for BRCA University of Pennsylvania Philadelphia Pennsylvania USA; ^4^ Department of Obstetrics Gynaecology and Newborn Health, the Royal Women's Hospital Melbourne Victoria Australia; ^5^ University of Sydney Sydney New South Wales Australia; ^6^ Department of Gynaecological Oncology Westmead Hospital Sydney New South Wales Australia; ^7^ Centre for Epidemiology and Biostatistics, Melbourne School of Population and Global Health The University of Melbourne Melbourne Victoria Australia; ^8^ Methods and Implementation Support for Clinical Health (MISCH) Research Hub, Faculty of Medicine, Dentistry and Health Sciences The University of Melbourne Melbourne Victoria Australia; ^9^ Department of Obstetrics and Gynecology, University Hospitals Cleveland Medical Center Case Western Reserve University Cleveland Ohio USA

**Keywords:** HRT, risk‐reducing salpingo‐oophorectomy, RRSO, sexual function, surgical menopause

## Abstract

**Objective:**

To determine the effect of surgical menopause (risk‐reducing salpingo‐oophorectomy, RRSO) on sexual function and the modifying effects of HRT.

**Design:**

Prospective observational study of women undergoing RRSO and age‐matched comparison group who retained their ovaries.

**Setting:**

High‐risk clinics and general population.

**Methods:**

Sexual function was measured at baseline, 3, 6, 12 and 24 months.

**Main Outcome Measures:**

Primary outcome was sexual function at 24 months using the Female Sexual Function Index (FSFI). Secondary outcomes included the Fallowfield Sexual Activity Questionnaire (SAQ) and Female Sexual Distress Scale‐Revised (FSDS‐R).

**Results:**

Baseline sexual function was similar between groups. At 24 months, sexual dysfunction increased from 19% to 42% after RRSO versus 24% to 29% in comparisons (Odds Ratio (OR) 1.9, 95% CI 0.7–5.1; *p* = 0.21). Compared to comparisons, sexual desire (−0.4, *p* = 0.02), arousal (−0.7, *p* < 0.001), lubrication (−0.6, *p* = 0.01) and satisfaction (−0.6, *p* < 0.001) were significantly reduced in the RRSO group. Sexual pain (−0.5, *p* = 0.05) and discomfort (−1.0, *p* < 0.001) increased after RRSO; sexual habit was unchanged. Sexual distress nearly quadrupled in the RRSO group (OR 3.7, 95% CI 1.6–9.0; *p* = 0.003). After RRSO, 61% commenced HRT. HRT was not associated with sexual function, activity or distress.

**Conclusions:**

Sexual dysfunction and distress increased after RRSO. Use of HRT was not associated with better sexual function.

## 1 Introduction

For women with pathogenic variants (PV) in the *BRCA1* or *BRCA2* genes, only risk‐reducing salpingo‐oophorectomy (RRSO) has been shown to prevent ovarian cancer deaths [1] and reduce all‐cause mortality [2]. The National Comprehensive Cancer Network recommends RRSO at age 40 for *BRCA1 PV* carriers and 45 for *BRCA2 PV* carriers [3], which will induce surgical menopause. Several prospective, cross‐sectional and retrospective studies have reported high rates of sexual dysfunction following RRSO [4, 5, 6].

Surgical menopause might impair sexual function due to vaginal dryness making sexual activity painful or uncomfortable [7] or loss of testosterone impacting sexual desire, arousal and satisfaction [8]. Changes in body image following mastectomy may also affect sexual function [9].

Understanding the impact of surgical menopause on sexual function is a patient priority [10], but whether HRT improves sexual function is uncertain. One prospective study reported a reduction in sexual pleasure and an increase in sexual discomfort up to 3 years after RRSO, mitigated but not prevented by HRT [11, 12]. A large (*n* = 577) patient preference study of salpingectomy alone versus RRSO reported reduced sexual function and increased distress which was modified by HRT use [13]. Similarly, a large (*n* = 817) cross‐sectional study reported increased overall sexual dysfunction (74%), hypoactive sexual desire disorder (73%), vaginal dryness (44%), reduced sexual satisfaction (41%), dyspareunia (28%) and orgasm difficulty (25%) after RRSO [9, 14] and that HRT improved dyspareunia [15].

We previously reported in WHAM that surgical menopause reduced sexual arousal, lubrication, orgasm and sexual pain at 12 months versus an age‐matched comparison group who preserved their ovaries [16]. However, we were unable to determine whether HRT mitigated this effect.

In the general population, a systematic review concluded that HRT has only a minor effect on sexual function [17] but topical oestrogen reduces vaginal dryness [18].

Most previous studies of sexual function after surgical menopause are cross‐sectional. In WHAM, we found that one third of women in the RRSO and comparison groups (31% vs. 37%) had elevated sexual distress at baseline, consistent with Australian population‐based data [19]. Hence, prospective studies are needed to determine the effects of surgical menopause on sexual function and efficacy of treatments such as HRT. Also, a comparison group of age‐matched women allows the effects of RRSO on sexual function to be compared with age‐matched women who retain their ovaries. The aim of this study was to prospectively measure sexual function, activity and distress 24 months after surgical menopause due to RRSO, compared to baseline and a comparison group who retained their ovaries, and explore how use of HRT affected sexual function.

## 2 Methods

Study participants included premenopausal women at elevated risk of ovarian cancer (pathogenic gene variants in *BRCA1/2, BRIP1, STK11* or Lynch syndrome or a strong family history) who were planning RRSO (RRSO group). Comparisons were age‐matched (within ±5 years) premenopausal women planning to retain their ovaries. All resided in Victoria or New South Wales (Australia), or Philadelphia, USA. Recruitment is described elsewhere [20]. In brief, premenopausal women at elevated risk of ovarian cancer and planning RRSO were referred to the study by clinicians and family cancer clinics. The comparison group was recruited through advertisements, social media, university and hospital newsletters and by clinic referrals. Eligible participants were aged 18 to 50 years with regular menstrual cycles (unless previous hysterectomy), serum FSH ≤ 15 IU/L and serum estradiol > 100 pmol/L. Those pregnant, lactating or taking anti‐oestrogen therapy in the previous 3 months were excluded. Women with abnormal vaginal bleeding, unable to complete the English language questionnaires, or unable to provide informed consent were excluded. Comparison group participants were excluded if they were planning to undergo oophorectomy or become pregnant during the 2‐year follow‐up period.

### 2.1 Patient Involvement

Patients were involved in planning the study. Previous research by the authors indicated that the nature, severity and management of symptoms after RRSO was a priority area for BRCA1/2 PV carriers [10].

### 2.2 Data Collection and Questionnaires Used

Demographic information, medical, surgical and gynaecological history, systemic and vaginal hormone therapy (HRT) and hormonal contraceptive use were documented at clinic visits at baseline, 3, 6, 12 and 24 months. The baseline visit was performed up to 8 weeks prior to surgery in the RRSO group or following eligibility screening for the comparison group. Other factors known to influence sexual function in premenopausal women, including depression, anxiety, psychotropic medication use, partner status and mastectomy were also documented [21]. The decision to take HRT and dose were clinically determined. All participants completed the Female Sexual Function Index (FSFI) [22], which has been validated in diverse groups of women including cancer survivors [23]. For women who were sexually active, this comprises 6 validated domains of sexual function reported for the past 4 weeks: desire (range 1.2–6; higher score indicating stronger desire), arousal (range 0–6; higher score indicating greater arousal), lubrication (range 0–6; higher score indicating more lubrication), orgasm (range 0–6; higher score indicating greater likelihood of orgasm), satisfaction (range 0.8–6; higher score indicating greater satisfaction) and pain during intercourse (range 0–6; higher score indicating less pain). A participant was considered sexually active within the 4 weeks prior to FSFI administration if they did not indicate responses of ‘no sexual activity’ for any of the 12 FSFI items Q7‐18, or responses of ‘did not attempt intercourse’ for any of the 3 FSFI items Q21‐23. The primary outcome for the WHAM study was female sexual dysfunction at 24 months, as measured by an FSFI overall score below 26.55 (range 2 to 36; higher scores indicating better functioning). Secondary outcomes include sexually‐related personal distress, determined when the total score on Female Sexual Distress Scale–Revised (FSDS‐R) [24] is ≥ 11 over a 30‐day recall period. Each of the 13 items in FSDS‐R are rated using a 5‐point scale (0–4; total score range 0–52 with higher scores indicating more distress). A score of ≥ 11 indicates sexually‐related personal distress with high reliability, discriminative ability and construct validity [24]. Sexual activity was measured by the Sexual Activity Questionnaire (SAQ) which measures pleasure (range 6–24; lower score indicating more pleasure), discomfort (range 2–10; lower score indicating more discomfort) and habit (range 1–4; lower score indicating more sexual activity) among women who were sexually active that is, those who responded ‘yes’ to being ‘involved in a sexual relationship’ to SAQ item 3 in Section 1, or those who did not respond ‘not at all’ to SAQ item 9 [25]. We did not use the Genitourinary Core Outcome measures [26] in this study as these were not published when the study was conducted.

### 2.3 Sample Size

The sample size was based on the available data pertaining to the proportion of premenopausal women with sexual dysfunction [20, 27, 28]. We estimated that 105 women planning RRSO and 105 comparisons would provide 80% power (two‐sided *α* = 0.05) to detect an increase in the proportion with sexual dysfunction at follow‐up from 24% in both groups at baseline to 45% in the RRSO group, allowing for 15% loss to follow‐up. This estimate assumed that the proportion of women with sexual dysfunction at baseline would not differ between the study groups and that the comparison group would have stable sexual function over the 24‐month follow‐up period [20].

### 2.4 Statistical Analyses

Descriptive statistics are presented as count and frequencies for categorical data and mean and standard deviation (SD) and/or median and interquartile range (25th to 75th percentile) for continuous data.

Calculation of the total FSFI score and most FSFI domain scores (except for the desire domain) requires participants to be sexually active (not having a zero score on any of the FSFI questionnaire items) [29]. Therefore, the analyses were limited to those who were sexually active as determined by FSFI and SAQ, women who completed the FSFI desire domain, and all women with FSDS‐R data. Number and proportions of sexually inactive women and missing outcomes were summarised by timepoint (Table S8). The primary outcome (sexual dysfunction) and binary secondary outcome (sexually‐related distress) were analysed using logistic regression. Mean differences in change in continuous secondary outcomes (FSFI domain scores, SAQ pleasure score, SAQ discomfort score, SAQ habit score and FSDS total score) from baseline to 24 months between RRSO and comparison groups were estimated using linear regression models. Ordinal logistic regression was fitted for SAQ habit score given the discrete and ordered nature. Both model 1 and model 2 included respective outcomes at baseline and age at baseline as per study design. Model 2 additionally adjusted for hormonal contraception use at baseline, previous breast cancer episodes pre‐baseline, and unilateral or bilateral mastectomy pre‐baseline. Due to non‐normality of the distribution of some domain scores at baseline, we incorporated bootstrapping with 1000 replications for continuous outcomes for inference.

For analyses investigating the impact of RRSO and HRT on sexual function, participants who underwent RRSO were further divided into 2 groups: (1) HRT users across 24 months and (2) non‐HRT users across 24 months. Tibolone (a synthetic product that binds to oestrogen, progesterone and androgen receptors) was included with the HRT users since a double‐blind study showed no difference in sexual function between tibolone and transdermal HRT [30]. Similar analyses were performed to investigate the change in outcomes from baseline to 24 months between HRT and non‐HRT users following RRSO, restricted to women in the RRSO group. Both model 1 and model 2 included respective outcomes at baseline and age at baseline as per study design. Model 2 additionally adjusted for symptoms of depression or anxiety at baseline, unilateral or bilateral mastectomy pre‐baseline and history of breast cancer at baseline. Secondary outcomes and the secondary comparison of HRT users after RRSO were not powered for. All reported 95% confidence intervals, 95% bootstrap bias‐corrected confidence intervals (hereafter all referred to as CIs) and *p*‐values are reported two‐sided, and no adjustment for multiple testing was performed. All analyses were performed using Stata/SE version 17.0 (StataCorp, College Station, TX, USA).

## 3 Results

**FIGURE 1:**
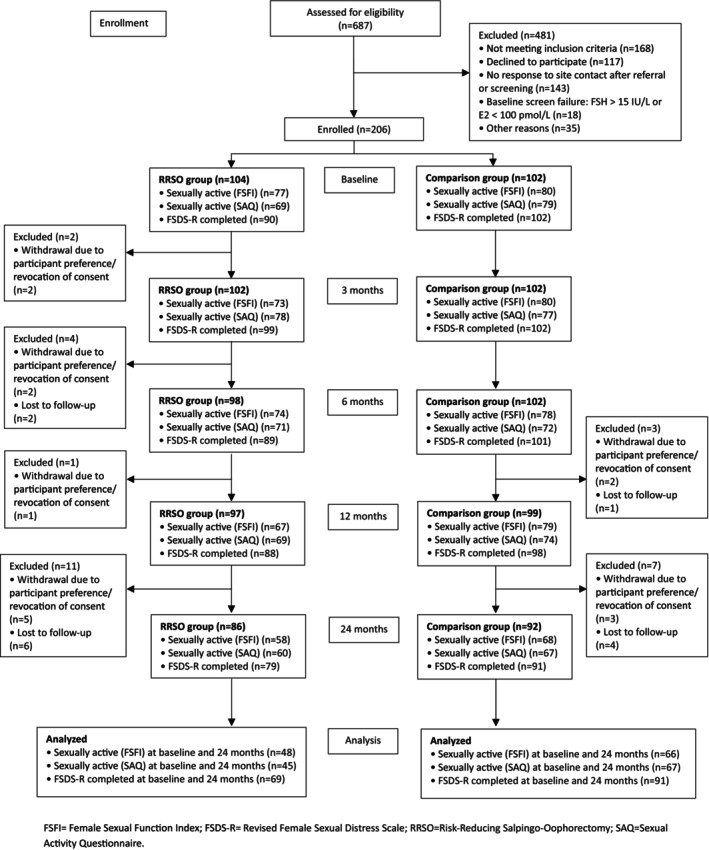
Flowchart of participant inclusion.

A total of 206 participants met entry criteria and were recruited into the study: *n* = 104 premenopausal women planning RRSO and *n* = 102 comparisons, of whom 86 (82.7%) and 92 (90.2%) completed the 24‐month follow‐up, respectively (Figure 1). The groups were matched in terms of age (mean 42.1 years in the RRSO group and 40.8 years in comparisons) (Table 1). Slightly more women in the comparison group were using hormonal contraception at baseline (34% [35/104] RRSO vs. 46% [47/102] comparison). More of the RRSO group had previous breast cancer (11% [11/104] vs. 2% [2/102]) and substantially more had previous unilateral or bilateral mastectomy (30% [31/103] vs. 5% [5/102]), either for breast cancer treatment or risk reduction, compared to the comparison group (Table 1). Most participants in the RRSO (76% [77/101]) and comparison groups (79% [80/101]) were sexually active at baseline, according to the FSFI. A small proportion of both groups had clinically significant depressive symptoms (15% [12/82] RRSO vs. 11% [11/101] comparison) or anxiety symptoms (12% [10/81] RRSO vs. 9% [9/101] comparison) at baseline.

**TABLE 1:** Characteristics of participants by study group.

	RRSO group			Comparison group	Total
	RRSO: HRT user^a^	RRSO: Non‐HRT user^a^	RRSO: Total		
	*N* = 63	*N* = 39	*N* = 104	*N* = 102	*N* = 206
**Recruitment site**					
The Royal Women's Hospital, Melbourne	34/63 (54%)	21/39 (54%)	56/104 (54%)	93/102 (91%)	149/206 (72%)
Other	29/63 (46%)	18/39 (46%)	48/104 (46%)	9/102 (9%)	57/206 (28%)
Age (years)	41.5 (3.7)	43.0 (4.6)	42.1 (4.1)	40.8 (5.8)	41.5 (5.0)
**Age (years) categorised**					
< 40	18/63 (29%)	12/39 (31%)	30/104 (29%)	43/102 (42%)	73/206 (35%)
≥ 40	45/63 (71%)	27/39 (69%)	74/104 (71%)	59/102 (58%)	133/206 (65%)
**Body Mass Index (kg/m** ^**2**^ **) categorised**					
Underweight (< 18.5)	1/63 (2%)	0/39 (0%)	1/103 (1%)	1/102 (1%)	2/205 (1%)
Normal (18.5 to < 25)	25/63 (40%)	16/39 (41%)	41/103 (40%)	52/102 (51%)	93/205 (45%)
Overweight (25 to < 30)	21/63 (33%)	11/39 (28%)	33/103 (32%)	31/102 (30%)	64/205 (31%)
Obese (≥ 30)	16/63 (25%)	12/39 (31%)	28/103 (27%)	18/102 (18%)	46/205 (22%)
**Ethnicity** ^b^					
European ancestry	49/63 (78%)	32/39 (82%)	82/104 (79%)	91/102 (89%)	173/206 (84%)
Other	14/63 (22%)	7/39 (18%)	22/104 (21%)	11/102 (11%)	33/206 (16%)
Education beyond high school	42/44 (95%)	22/27 (81%)	64/71 (90%)	91/94 (97%)	155/165 (94%)
**Relationship status** ^c^					
Married/Defacto	53/63 (84%)	34/39 (87%)	87/104 (84%)	80/102 (78%)	167/206 (81%)
Other	10/63 (16%)	5/39 (13%)	17/104 (16%)	22/102 (22%)	39/206 (19%)
**Smoking status**					
Non‐smoker	43/63 (68%)	17/39 (44%)	61/103 (59%)	63/102 (62%)	124/205 (60%)
Ex‐smoker	15/63 (24%)	19/39 (49%)	34/103 (33%)	33/102 (32%)	67/205 (33%)
Current smoker	5/63 (8%)	3/39 (8%)	8/103 (8%)	6/102 (6%)	14/205 (7%)
Alcohol consumer	53/63 (84%)	37/39 (95%)	91/103 (88%)	95/102 (93%)	186/205 (91%)
Hysterectomy pre‐baseline	0/63 (0%)	1/38 (3%)	1/103 (1%)	3/102 (3%)	4/205 (2%)
**Parity**					
Non‐parous	13/63 (21%)	6/39 (15%)	19/103 (18%)	28/102 (27%)	47/205 (23%)
Parous	50/63 (79%)	33/39 (85%)	84/103 (82%)	74/102 (73%)	158/205 (77%)
Hormonal contraception	22/63 (35%)	12/39 (31%)	35/104 (34%)	47/102 (46%)	82/206 (40%)
Unilateral or bilateral mastectomy pre‐baseline	18/63 (29%)	13/39 (33%)	31/103 (30%)	5/102 (5%)	36/205 (18%)
Progestogen only oral contraception	1/63 (2%)	0/39 (0%)	1/103 (1%)	1/102 (1%)	2/205 (1%)
Depo‐medroxy progesterone acetate or contraceptive implant	2/63 (3%)	2/39 (5%)	4/103 (4%)	5/102 (5%)	9/205 (4%)
LNG‐IUD only in situ	11/63 (17%)	5/39 (13%)	17/103 (17%)	22/102 (22%)	39/205 (19%)
Systemic oestrogen contraception	14/63 (22%)	7/39 (18%)	21/104 (20%)	21/102 (21%)	42/206 (20%)
Sexually active (FSFI)^d^	47/62 (76%)	30/38 (79%)	77/101 (76%)	80/101 (79%)	157/202 (78%)
Sexually active (SAQ)^e^	46/59 (78%)	23/30 (77%)	69/90 (77%)	79/102 (77%)	148/192 (77%)
Depression symptoms at baseline	8/54 (15%)	4/27 (15%)	12/82 (15%)	11/101 (11%)	23/183 (13%)
Anxiety symptoms at baseline	7/53 (13%)	3/27 (11%)	10/81 (12%)	9/101 (9%)	19/182 (10%)
Anti‐depressant use at baseline	6/63 (10%)	8/39 (21%)	14/102 (14%)	12/102 (12%)	26/204 (13%)
Breast cancer pre‐baseline^f^	5/63 (8%)	6/39 (15%)	11/104 (11%)	2/102 (2%)	13/206 (6%)
Bothered by night sweats	12/63 (19%)	9/38 (24%)	21/102 (21%)	25/102 (25%)	46/204 (23%)
Good sleep quality^g^	34/62 (55%)	15/37 (41%)	50/100 (50%)	58/102 (57%)	108/202 (53%)

*Note:* Data are presented as mean (standard deviation) for continuous measures and n/N (%) for categorical measures.

Abbreviations: FSFI, Female Sexual Function Index; HRT, Hormone Replacement Therapy; LNG‐IUD, levonorgestrel intra‐uterine device; RRSO, Risk‐Reducing Salpingo‐Oophorectomy; SAQ, Sexual Activity Questionnaire.

^a^ The HRT use of two RRSO participants was unknown.

^b^ Other ethnicities include Asian, Eurasian, Euro‐Caribbean, Latin‐American and unknown.

^c^ Other relationship statuses include boy/girlfriend, casual, no partner and unknown.

^d^ Percentage out of N who responded to the FSFI at baseline. A participant was considered sexually active within the 4 weeks prior to FSFI administration if they did not indicate responses of ‘no sexual activity’ for any of the 12 FSFI items Q7‐18 or responses of ‘did not attempt intercourse’ for any of the 3 FSFI items Q21‐23.

^e^ Percentage out of N who responded to the SAQ at baseline. A participant was considered sexually active if they responded to being ‘involved in a sexual relationship’ (i.e., patients who responded ‘Yes’ to question 3 [Do you engage in sexual activity with anyone at the moment?] of Section 1 of the SAQ), or they did not indicate ‘not at all’ to question 9 [How often did you engage in sexual activity this month?] whenever Section 1 of the SAQ was administered to participants.

^f^ Both comparison participants were BRCA1 carriers.

^g^ Good sleep quality was determined if total PSQI (Pittsburgh Sleep Quality Index) score ≤ 5.

### 3.1 Change in Sexual Function (FSFI) Following RRSO

Among all those with data at baseline and 24 months, 48/75 (64%) of the RRSO group and 66/91 (73%) of the comparison group reported being sexually active in the FSFI assessments at both time‐points (Tables 2 and S5), and the proportion in each group was similar at baseline (76% [77/101] RRSO vs. 79% [80/101] comparisons) (Table 1). The proportion of participants meeting criteria for sexual dysfunction increased from 19% (9/48) to 42% (20/48) in the RRSO group and 24% (16/66) to 29% (19/66) in the comparison group (OR 1.9; CI 0.7 to 5.1; *p* = 0.21), but this difference was not statistically significant. The FSFI total score and its 5 subdomains (desire, arousal, lubrication, satisfaction and pain) indicated strong evidence of worse sexual function outcomes in the RRSO group when compared to the comparisons at 24 months (Table 2). Individual questions from the FSFI are summarised over time in Tables S2 and S7.

**TABLE 2:** Between‐group differences in RRSO vs. Comparison group at 24 months in participants sexually active at both baseline and 24 months^a^.

Outcome	Baseline^b^		24 months^b^		Between‐group difference (RRSO vs. Comparison)					
	RRSO	Comparison	RRSO	Comparison	Model 1^c^			Model 2^d^		
					*N*	Estimate (95% CI)^e^	*p*	*N*	Estimate (95% CI)^e^	*p*
**FSFI**	***N* = 48**	***N* = 66**	***N* = 48**	***N* = 66**						
*Primary outcome*										
Sexual dysfunction	9/48 (19%)	16/66 (24%)	20/48 (42%)	19/66 (29%)	114	2.3 (0.9, 5.7)	0.07	114	1.9 (0.7, 5.1)	0.21
*Secondary outcomes*										
Overall score	30.7 (27.5–33.0)	29.6 (26.7–31.7)	27.5 (22.7–30.6)	30.6 (24.9–32.4)	114	−3.4 (−5.1, −1.6)	< 0.001	114	−3.2 (−5.2, −1.3)	< 0.001
Desire Score (all participants)	3.6 (2.4–4.2)	3.6 (2.4–3.6)	2.7 (1.8–3.6)	3.0 (2.4–4.2)	168	−0.4 (−0.8, −0.1)	0.01	168	−0.5 (−0.8, −0.2)	< 0.001
Desire Score^f^	3.6 (3.0–4.2)	3.6 (2.4–3.6)	3.0 (2.4–3.6)	3.6 (2.4–4.2)	114	−0.5 (−0.8, −0.1)	0.01	114	−0.4 (−0.8, −0.1)	0.02
Arousal Score	4.8 (4.2–5.7)	4.8 (4.5–5.7)	4.0 (3.1–5.1)	5.1 (3.9–5.7)	114	−0.8 (−1.2, −0.4)	< 0.001	114	−0.7 (−1.1, −0.3)	< 0.001
Lubrication Score	5.7 (5.4–6.0)	5.7 (5.4–6.0)	5.4 (4.5–5.7)	5.8 (4.8–6.0)	114	−0.5 (−0.9, −0.1)	0.007	114	−0.6 (−1.0, −0.1)	0.01
Orgasm Score	5.6 (4.8–6.0)	5.4 (4.4–6.0)	4.8 (3.4–6.0)	5.6 (4.4–6.0)	114	−0.5 (−1.0, 0.0)	0.06	114	−0.5 (−1.0, 0.1)	0.10
Satisfaction Score	5.6 (4.8–6.0)	5.0 (4.0–5.6)	4.8 (3.2–5.6)	5.2 (4.8–5.6)	111	−0.7 (−1.1, −0.3)	< 0.001	111	−0.6 (−1.0, −0.2)	< 0.001
Pain Score	6.0 (5.6–6.0)	6.0 (5.2–6.0)	6.0 (5.2–6.0)	6.0 (5.6–6.0)	114	−0.4 (−0.8, 0.0)	0.03	114	−0.5 (−0.9, 0.0)	0.05
**SAQ**	***N* = 45**	***N* = 67**	***N* = 45**	***N* = 67**						
Pleasure score	10.0 (8.0–12.0)	12.0 (9.0–14.0)	13.0 (9.0–16.0)	11.5 (9.0–15.0)	110	1.2 (−0.2, 2.6)	0.09	110	1.3 (−0.2, 2.8)	0.08
Discomfort score	8.0 (7.0–8.0)	7.0 (6.0–8.0)	7.0 (5.0–8.0)	8.0 (7.0–8.0)	106	−0.9 (−1.5, −0.3)	< 0.001	106	−1.0 (−1.6, −0.3)	< 0.001
Habit score^g^	3.0 (3.0–3.0)	3.0 (3.0–3.0)	3.0 (3.0–4.0)	3.0 (3.0–4.0)	112	1.0 (0.5, 2.1)	0.96	112	1.2 (0.5, 2.7)	0.65
**FSDS‐R**	***N* = 69**	***N* = 91**	***N* = 69**	***N* = 91**						
Total Score	5.4 (0.0–13.0)	7.0 (2.0–14.0)	10.0 (1.0–19.0)	4.0 (1.0–13.0)	160	4.5 (1.6, 7.4)	< 0.001	160	4.8 (1.3, 8.4)	0.01
Sexually related distress	21/69 (30%)	33/91 (36%)	32/69 (46%)	26/91 (29%)	160	3.5 (1.5, 8.0)	0.003	160	3.7 (1.6, 9.0)	0.003

*Note:* Data are presented as median (IQR) for continuous measures, and n/N (%) for categorical measures at baseline and 24 months.

Abbreviations: CI, confidence interval; FSDS‐*R*, Revised Female Sexual Distress Scale; FSFI, Female Sexual Function Index; IQR, interquartile range; *N*, Number of patients included in the model; RRSO, risk‐reducing salpingo‐oophowrectomy; SAQ, Sexual Activity Questionnaire.

^a^ Participants who were sexually active in FSFI/SAQ at both baseline and 24 months were included in analysis for FSFI/SAQ outcomes respectively. Participants with available FSDS‐R outcomes at both baseline and 24 months were included in analysis for FSDS‐R outcomes.

^b^ Descriptive statistics for baseline and 24 months are based on the available complete case data in participants sexually active at baseline and 24 months respectively.

^c^ Model 1 included respective outcomes at baseline and age at baseline.

^d^ Model 2 was adjusted for respective outcome at baseline, age at baseline, hormonal contraception use at baseline, unilateral or bilateral mastectomy at pre‐baseline and breast cancer episode pre‐baseline.

^e^ Estimated odds ratio (95% CI) is presented for categorical outcomes. Estimated mean difference (95% CI) is presented for continuous outcomes. Estimated proportional odds ratio (95% CI) is presented for SAQ Total habit score.

^f^ Sensitivity analysis for FSFI Desire domain, which included participants who were sexually active in FSFI at both baseline and 24 months.

^g^ Ordinal logistic regression models were fitted for SAQ Total habit score.

### 3.2 Change in Sexual Activity (SAQ) Following RRSO

Among all those with data at baseline and 24 months, 45/69 (76%) of the RRSO group and 67/92 (73%) of the comparison group reported being sexually active, as measured by the SAQ at both time‐points (Tables 2 and S6). The SAQ total pleasure and discomfort scores indicated worse outcomes at 24 months compared to baseline; that is, mean between‐group difference of 1.3 (CI −0.2 to 2.8, *p* = 0.08) for pleasure, and −1.0 (CI −1.6 to −0.3, *p* < 0.001) for discomfort score. No change in sexual habit in either group was observed (mean between‐group difference 1.2; CI 0.5 to 2.7, *p* = 0.65) (Table 2). Individual questions of the SAQ are summarised over time in Tables S3 and S7.

### 3.3 Change in Sexually‐Related Personal Distress Following RRSO

At baseline, there was no significant difference between the groups, with around one third of participants reporting sexual distress (RRSO 30% [21/69] vs. comparisons 36% [33/91]). After RRSO, the proportion of women with sexual distress increased to 46% (32/69) at 24 months compared to baseline but did not increase in the comparison group, 29% (26/91) (OR 3.7; CI 1.6 to 9.0, *p* = 0.003) (Table 2). Individual questions of the FSDS‐R are summarised over time in Tables S4 and S7.

### 3.4 Use of Hormone Replacement Therapy

No participants in either group were using HRT at baseline, and none of the comparison group were using HRT at 24 months (Table S1). Following RRSO, 61% (63/104) commenced HRT. The indication (e.g., as prevention against osteoporosis or as treatment of menopausal symptoms) was not recorded. The oestrogen dose of HRT was not standardised in this study but around half (54%, 34/63) were taking doses equivalent to ≥ 50 mg estradiol/day (Table S1). Based on data collected at each visit, 84% (42/50) were adherent to HRT for at least 70% of the 24‐month follow‐up period. A range of transdermal and oral HRT preparations were used (Table S1). Because around one third of women underwent hysterectomy at the time of RRSO, these participants (17/63) used oestrogen‐only HRT. Only 8 (8%) used vaginal oestrogen after RRSO. Of those, 5 also used systemic HRT and 3 used vaginal oestrogen alone. Only 4% (*n* = 4) took systemic testosterone after RRSO. Comparison of the baseline characteristics of RRSO participants who went on to be HRT users or not, indicated that symptoms of depression and anxiety were similar in both groups, while slightly less HRT users were bothered by night sweats (19% [12/63] vs. 24% [9/38]) and more HRT users reported good quality sleep (55% [34/62] vs. 41% [15/37]) (Table 1). Less HRT users (8% [5/63] vs. 15% [6/39]) had previous breast cancer, but similar proportions had previous unilateral or bilateral mastectomy.

### 3.5 HRT and Sexual Function

Among those who were sexually active at baseline and 24 months in the RRSO group, there was a non‐statistically significant difference in the proportion with sexual dysfunction (21% (6/28) to 36% (10/28) in those who took HRT and 15% (3/20) to 50% (10/20) in non‐HRT users (OR 1.1; CI 0.2 to 6.3; *p* = 0.89)). There was no evidence for a change in secondary outcomes from baseline to 24 months between the HRT users and non‐users after RRSO (Table 3).

**TABLE 3:** Between‐group differences by HRT use at 24 months in participants sexually active at baseline and 24 months in RRSO group^a^.

Outcome	Baseline^b^		24 months^b^		Between‐group difference (HRT vs. Non‐HRT user)					
	HRT user	Non‐HRT user	HRT user	Non‐HRT user	Model 1^c^			Model 2^d^		
					*N*	Estimate (95% CI)^e^	*p*	*N*	Estimate (95% CI)^e^	*p*
**FSFI**	***N* = 28**	***N* = 20**	***N* = 28**	***N* = 20**						
*Primary outcome*										
Sexual dysfunction	6/28 (21%)	3/20 (15%)	10/28 (36%)	10/20 (50%)	48	0.5 (0.1, 1.6)	0.24	37	1.1 (0.2, 6.3)	0.89
*Secondary outcomes*										
Overall score	29.4 (26.7–32.9)	31.3 (29.4–33.0)	28.1 (24.8–30.9)	25.8 (20.4–29.4)	48	3.3 (0.0, 6.6)	0.05	37	1.9 (−2.8, 6.7)	0.43
Desire Score (all participants)	3.6 (2.4–3.6)	3.6 (2.4–4.2)	3.0 (2.4–3.6)	2.4 (1.2–3.6)	76	0.4 (−0.1, 0.9)	0.10	60	0.4 (−0.2, 1.0)	0.20
Desire Score^f^	3.6 (2.7–4.2)	3.6 (3.3–4.5)	3.6 (2.4–3.6)	2.4 (2.4–3.6)	48	0.7 (0.1, 1.3)	0.02	37	0.6 (−0.2, 1.4)	0.15
Arousal Score	4.8 (4.2–5.2)	5.4 (4.5–5.7)	4.3 (3.6–5.1)	3.3 (2.7–5.0)	48	0.9 (0.2, 1.5)	0.01	37	0.8 (−0.4, 2.0)	0.17
Lubrication Score	5.7 (5.4–6.0)	6.0 (5.5–6.0)	5.7 (4.8–5.8)	4.7 (3.9–5.5)	48	0.8 (0.2, 1.4)	0.01	37	0.5 (−0.3, 1.3)	0.23
Orgasm Score	5.4 (4.2–5.8)	6.0 (5.0–6.0)	4.8 (4.0–5.6)	4.4 (2.6–6.0)	48	0.9 (0.0, 1.7)	0.05	37	0.4 (−1.1, 1.9)	0.60
Satisfaction Score	5.6 (4.8–6.0)	5.4 (5.0–6.0)	4.8 (3.4–5.6)	4.8 (3.2–5.6)	47	−0.1 (−0.8, 0.6)	0.83	36	−0.4 (−1.3, 0.5)	0.41
Pain Score	6.0 (5.6–6.0)	6.0 (5.8–6.0)	6.0 (5.6–6.0)	5.6 (5.2–6.0)	48	0.2 (−0.5, 1.0)	0.53	37	−0.2 (−0.9, 0.5)	0.56
**SAQ**	***N* = 28**	***N* = 17**	***N* = 28**	***N* = 17**						
Pleasure score	10.5 (8.5–13.0)	10.0 (8.0–11.0)	13.0 (9.0–17.0)	13.5 (10.0–16.0)	44	−0.7 (−3.2, 1.8)	0.57	37	−1.2 (−4.4, 1.9)	0.45
Discomfort score	8.0 (7.0–8.0)	8.0 (7.0–8.0)	7.0 (6.0–8.0)	6.5 (5.0–8.0)	41	0.6 (−0.4, 1.7)	0.23	36	0.3 (−0.9, 1.4)	0.65
Habit score^g^	3.0 (2.0–3.0)	3.0 (3.0–3.0)	3.0 (3.0–4.0)	3.0 (2.0–3.0)	45	2.3 (0.7, 7.6)	0.17	38	1.6 (0.4, 6.7)	0.54
**FSDS‐R**	***N* = 45**	***N* = 24**	***N* = 45**	***N* = 24**						
Total score	5.4 (2.0–13.0)	5.5 (0.0–11.5)	10.0 (2.0–17.0)	10.0 (0.0–19.0)	69	−0.6 (−5.8, 4.6)	0.82	61	−0.9 (−7.3, 5.5)	0.78
Sexually related distress	15/45 (33%)	6/24 (25%)	20/45 (44%)	12/24 (50%)	69	0.6 (0.2, 1.8)	0.31	61	0.5 (0.1, 2.1)	0.33

*Note:* Data are presented as median (IQR) for continuous measures and n/N (%) for categorical measures at baseline and 24 months.

Abbreviations: CI, confidence interval; HRT, Hormone Replacement Therapy; IQR, Interquartile range; *N*, Number of patients included in the model; RRSO, risk‐reducing salpingo‐oophorectomy.

^a^ Participants in RRSO group who were sexually active in FSFI/SAQ at both baseline and 24 months were included in analysis for FSFI/SAQ outcomes respectively. Participants in RRSO group with available FSDS‐R outcomes at both baseline and 24 months were included in analysis for FSDS‐R outcomes.

^b^ Descriptive statistics for baseline and 24 months are based on the available complete case data in participants sexually active at baseline and 24 months respectively.

^c^ Model 1 included respective outcomes at baseline and age at baseline.

^d^ Model 2 was adjusted for respective outcome at baseline, age at baseline, symptoms of depression or anxiety at baseline, unilateral or bilateral mastectomy at pre‐baseline and breast cancer episode at baseline.

^e^ Estimated odds ratio (95% CI) is presented for categorical outcomes. Estimated mean difference (95% CI) is presented for continuous outcomes. Estimated proportional odds ratio (95% CI) is presented for SAQ Total habit score.

^f^ Sensitivity analysis for FSFI Desire domain which included participants in RRSO who were sexually active in FSFI at both baseline and 24 months.

^g^ Ordinal logistic regression models were fitted for SAQ Total habit score.

## 4 Discussion

### 4.1 Main Findings

In this large prospective study of premenopausal women undergoing surgical menopause as RRSO and a comparison group who retained their ovaries, RRSO resulted in a non‐significant (OR 1.9; CI 0.7 to 5.1; *p* = 0.21) increase in sexual dysfunction at 24 months compared to baseline (prior to RRSO). All aspects of sexual function (desire, arousal, lubrication, satisfaction and pain) worsened 24 months after RRSO versus the comparison group. Similarly, whilst sexual habit was generally maintained, RRSO increased discomfort and pain and reduced sexual satisfaction at 24 months. Whilst the proportion with sexual dysfunction doubled following RRSO, this did not reach statistical significance, likely due to limited sample size. Sexual distress increased almost four‐fold after RRSO, but this increase did not differ between those who initiated HRT and those who did not.

Our findings are generally consistent with the published prospective [11, 12] and cross‐sectional data on sexual function after RRSO [9, 14]. Similarly, cross‐sectional studies after surgical menopause in the general population suggest that sexual dysfunction is common, but also do not have the benefit of a comparison group as in WHAM [31]. However, our findings suggest that RRSO has a clinically significant and persistent adverse effect on sexual function which may require longer‐term follow‐up and proactive management. We previously reported that whilst high‐risk women and clinicians are well informed about the efficacy of RRSO to prevent ovarian cancer, they are much less aware of the consequences and effective management of surgical menopause [32]. Also, that concerns about surgical menopause may discourage women from potentially life‐saving RRSO [10, 32]. Counselling about the effects of RRSO on sexual function is often sub‐optimal [33]. Effective management pre‐ and post‐RRSO should include the likely consequences of surgical menopause for sexual function and potential management strategies [34]. However, more information is needed about the optimal way to manage the adverse sexual consequences of premenopausal RRSO, since treatment with average doses of HRT does not appear to be sufficient. Around one third of participants were under age 40 years at RRSO. 2024 European Society of Human Reproduction (ESHRE) guidelines recommend higher oestrogen doses (100 mcg or 2 mg oral oestrogen) for these women [35]. It is possible that higher oestrogen doses may have been beneficial. However, use of MHT was clinically determined in WHAM so it was not possible to dictate the dose used. A small prospective cohort study in 2024 reported that MHT improved sexual function after RRSO (vs. non‐users) but only half were premenopausal at baseline and half had previous breast cancer, limiting comparison with WHAM data [36].

Around 60% of participants elected to take HRT after RRSO, a similar uptake to that reported in other studies [37]. Use of systemic HRT is encouraged following surgical menopause for symptomatic women without contraindications such as previous breast cancer [34]. However, the optimum dose and duration of HRT in *BRCA1/2* pathogenic variant carriers are uncertain [38]. There are several mechanisms by which RRSO may adversely affect sexual function. Loss of ovarian oestrogen may directly affect the urogenital tract leading to genitourinary symptoms such as pain with sex, vulvovaginal dryness and discomfort, along with discomfort/pain when urinating [39]. Evidence supports the use of oestrogen to improve some of these symptoms, particularly vaginal dryness [40]. However, despite the use of HRT, sexual dysfunction and distress increased after RRSO. In the general population, vaginal oestrogen is recommended for genitourinary symptoms associated with menopause [41]. Very few WHAM participants used vaginal oestrogen, which may have contributed to the sexual discomfort observed after RRSO. Future studies should consider trialling vaginal oestrogen for sexual discomfort in this population. The role of testosterone for sexual difficulties following RRSO also needs further investigation.

### 4.2 Strengths and Limitations

#### 4.2.1 Strengths

A relatively large sample size and prospective design including a similar aged comparison group who retained their ovaries. A key strength of prospective studies is their ability to collect baseline measures. In WHAM, we observed that more than one quarter of participants (31% RRSO, 37% comparisons) had elevated sexual distress at baseline. Hence, prospective studies are needed to demonstrate any additional effect of RRSO on sexual function and the efficacy of treatments such as HRT. A further strength is that all participants were premenopausal at baseline, as distinct from several other cohort studies where this was not established. Also, the use of validated questionnaires that measured several domains of sexual function and sexually‐related personal distress. Data on initiation, dose and delivery of HRT were collected prospectively. We selected the FSFI sexual dysfunction as the primary outcome because this is the most widely used, validated sexual function questionnaire in women.

#### 4.2.2 Limitations

The study was planned for the primary objective to compare sexual function (FSFI) between the RRSO and comparison group at 24 months in all eligible participants regardless of sexual activity. Unfortunately, the FSFI is only valid in women who are sexually active and there is no validated method to measure sexual function in those who are inactive. Excluding those who were not sexually active reduced the sample size for this study, the primary outcome for WHAM. Hence, our sexual function findings cannot be generalised to women who are not sexually active or to determine how HRT affected sexual function. Similarly, only those able to speak English were included and findings may differ in other groups. Factors such as psychotropic medication use, previous breast cancer and/or mastectomy, sexual inactivity and infertility treatment may contribute to sexually related distress [42]. We did not evaluate relationship satisfaction, which may predict ongoing sexual activity after RRSO despite symptoms such as vaginal dryness [43]. Use of HRT was not randomised in this study and we recognise that those who chose to use HRT may have differed from those who did not (e.g., non‐users who have a personal history of breast cancer). Additionally, HRT dose varied between participants. Those with worse menopausal symptoms may have been more likely to take HRT and our study was not powered to detect the effects of HRT use on sexual function. Our analysis includes multiple comparisons which increases the likelihood of false positive findings. Sexual function in women is complex and may be impacted by knowledge of *BRCA1/2* status in addition to the effects of breast cancer and mastectomy [44]. However, published studies report mixed findings about whether mastectomy +/− reconstruction affects sexual function [44, 45].

## 5 Interpretation

This study confirms findings from cross‐sectional and some longitudinal reports that surgical menopause following RRSO adversely affects sexual function and increases distress. We found no association between sexual function and systemic HRT use, suggesting that other interventions may be needed to preserve and improve sexual function after RRSO.

## 6 Conclusion

Surgical menopause as premenopausal RRSO adversely affected several aspects of sexual function at 24 months with increased sexual dysfunction and discomfort and decreased sexual pleasure. High risk women and their clinicians should be aware of the likely adverse effects on sexual function that may not be fully resolved by HRT. Because sexual pain increases after RRSO, clinicians should consider offering vaginal oestrogen, even for those using systemic HRT [34].

## Author Contributions

M.H. led the WHAM study and data interpretation, drafted the manuscript and is responsible for the final version. T.T.‐B., S.M.D. and A.B. recruited participants and contributed to data interpretation and manuscript writing. E.O.K. managed the WHAM study and contributed to data interpretation and manuscript writing. S.B. and P.L. led the data analysis. S.K. contributed expert knowledge on sexual function in women.

## Funding

Supported in Australia by National Health and Medical Research Council (NHMRC) #APP1048023, The Royal Women's Hospital, The Women's Foundation, Australia New Zealand Gynaecological Oncology Group (ANZGOG) and Westmead Hospital Familial Cancer Service. US support: Basser Center for BRCA and Susan G. Komen Organisation (SAC150003).

## Disclosure

Clinical Trials Registration: Australian New Zealand Clinical Trials Registry (anzctr.org.au); Identifier #: ACTRN12615000082505; URL: https://www.anzctr.org.au/Trial/Registration/TrialReview.aspx?id=363554&isReview=true. Prior Presentations/Publications: 12 months data on sexual function from WHAM was previously published [16].

## Ethics Statement

The study received ethics approval from the Human Research Ethics Committees (HRECs, Australia) or Institutional Review Boards (IRBs, USA) that were responsible for each recruitment site. These approvals included: Peter MacCallum Cancer Centre HREC, Melbourne, Australia (HREC/12/PMCC/24, Date 05/12/2017), the Prince of Wales Hospital HREC, Sydney, Australia (HREC/13/POWH/61, Date 02/05/2018), the Royal Prince Alfred Hospital HREC, Sydney, Australia (X14/0396, 10/04/2018) and the University of Pennsylvania IRB, Philadelphia, USA (CTSRMC/UPCC/01813, 14/02/2013). All participants provided written informed consent prior to any data collection.

## Conflicts of Interest

M.H. is an editor for the Cochrane Collaboration Group and was a topic expert for the National Institute of Care and Clinical Excellence (NICE) UK menopause guidelines and for the World Health Organisation prevention of dementia taskforce. She has received funding from the National Health and Medical Research Centre (NHMRC) and Medical Research Future Fund (MRFF). S.M.D. is the Executive Director of the Basser Center for BRCA and has received honoraria from AstraZeneca outside of the submitted work. E.O.K., P.L., T.T.‐B., S.B., A.B. and S.K. have no competing interests to declare. The funding organisations have not had any role in the conduct of the research nor the preparation and submission of this article, and there are no other relationships or activities that could appear to have influenced the submitted work.

## Supporting information


**Table S1:** Hormone Replacement Therapy use between baseline and 24 months in RRSO group.

**Table S2:** Descriptive statistics of Female Sexual Function Index (FSFI) in sexually active participants by timepoint and study group.

**Table S3:** Descriptive statistics of Sexual Activity Questionnaire (SAQ) in sexually active participants by timepoint and study group.

**Table S4:** Descriptive statistics of Revised Female Sexual Distress Scale in all participants by timepoint and study group.

**Table S5:** Baseline and 24‐month follow‐up sexual activity status in Female Sexual Function Index (FSFI).

**Table S6:** Baseline and 24‐month follow‐up sexual activity status in Sexual Activity Questionnaire (SAQ).

**Table S7:** Descriptive statistics of outcomes in sexually active participants by study group and timepoint.

**Table S8:** Missing data patterns by timepoint.

## Data Availability

The data that support the findings of this study are available on request from the corresponding author. The data are not publicly available due to privacy or ethical restrictions.
