# Supplementary material for: Sexual Function, Activity and Distress 24 Months After Surgical Menopause: What Happens After Menopause (WHAM)—A Prospective Controlled Study
Source: BJOG. 2026 Jan 22;133(6):1188–99. doi: 10.1111/1471-0528.70158 (PMC13040429; doi:10.1111/1471-0528.70158)
Supplement: Supplementary file 1 — Table S1: Hormone Replacement Therapy use between baseline and 24 months in RRSO group. [file BJO-133-1188-s003.docx]

**S1. Hormone Replacement Therapy use between baseline and 24 months in RRSO group.**

|  | **RRSO: HRT use** | | | **RRSO:**  **Total** |
| --- | --- | --- | --- | --- |
|  | **HRT**  **user** | **Non-HRT user** | **HRT use unknown** |  |
|  | **N=63** | **N=39** | **N=2** | **N=104** |
| Study period started using HRT |  |  |  |  |
| Not used | ·· | 39 (100%) | ·· | 39 (38%) |
| Used HRT | 63 (100%) | ·· | ·· | 63 (61%) |
| Between RRSO and 3 months | 50 (79%) | ·· | ·· | 50 (48%) |
| Between 3 months and 6 months after RRSO | 6 (10%) | ·· | ·· | 6 (6%) |
| Between 6 months and 12 months after RRSO | 5 (8%) | ·· | ·· | 5 (5%) |
| Between 12 months and 24 months after RRSO | 2 (3%) | ·· | ·· | 2 (2%) |
| Unknown | ·· | ·· | 2 (100%) | 2 (2%) |
| HRT use within first week after RRSO |  |  |  |  |
| No | 28 (44%) | 39 (100%) | ·· | 67 (64%) |
| Yes | 34 (54%) | ·· | ·· | 34 (33%) |
| Unknown | 1 (2%) | ·· | 2 (100%) | 3 (3%) |
| Used HRT (estrogen only or combined estrogen and progestin) between RRSO and 24 months |  |  |  |  |
| Not used | ·· | 39 (100%) | ·· | 39 (38%) |
| Estrogen Only Users (Total) | 17 (27%) | ·· | ·· | 17 (16%) |
| Estrogen Oral (one drug) | 1 (2%) | ·· | ·· | 1 (1%) |
| Estrogen Transdermal (one drug) | 15 (24%) | ·· | ·· | 15 (14%) |
| Other estrogen HRT ^a^ | 1 (2%) | ·· | ·· | 1 (1%) |
| Combination estrogen and progestin users (Total) | 31 (49%) | ·· | ·· | 31 (30%) |
| Combination estrogen and progestin oral (one drug) | 2 (3%) | ·· | ·· | 2 (2%) |
| Combination estrogen and progestin transdermal (one drug) | 1 (2%) | ·· | ·· | 1 (1%) |
| Oral estrogen and oral progestin or IUD (two drugs) | 13 (21%) | ·· | ·· | 13 (13%) |
| Transdermal estrogen and oral progestin or IUD (two drugs) | 9 (14%) | ·· | ·· | 9 (9%) |
| Other Combination estrogen and progestin HRT | 6 (10%) | ·· | ·· | 6 (6%) |
| Tibolone Users (Total) | 2 (3%) | ·· | ·· | 2 (2%) |
| Unknown ^b^ | 13 (21%) | ·· | 2 (100%) | 15 (14%) |
| HRT formulation prescribed dosage between surgery and 24 months ^c^ |  |  |  |  |
| Not used | ·· | 39 (100%) | ·· | 39 (38%) |
| Estrogen Only Users |  |  |  |  |
| <50 μg | 5 (8%) | ·· | ·· | 5 (5%) |
| ≥50 and ≤75 μg | 9 (14%) | ·· | ·· | 9 (9%) |
| >75 μg | 2 (3%) | ·· | ·· | 2 (2%) |
| Dosage unknown | 1 (2%) | ·· | ·· | 1 (1%) |
| Combined estrogen and progestin users |  |  |  |  |
| <50 μg | 8 (13%) | ·· | ·· | 8 (8%) |
| ≥50 and ≤75 μg | 10 (16%) | ·· | ·· | 10 (10%) |
| >75 μg | 13 (21%) | ·· | ·· | 13 (13%) |
| Tibolone users (2.5 mg) | 2 (3%) | ·· | ·· | 2 (2%) |
| Unknown ^b^ | 13 (21%) | ·· | 2 (100%) | 15 (14%) |
| Level of adherence (%) ^d^ | 97 (82-100); n=50 | ·· | ·· | 97 (82-100); n=50 |
| < 70% | 8 (16%) |  | ·· | 8 (16%) |
| ≥ 70% | 42 (84%) |  | ·· | 42 (84%) |
| Estrogen dosing behaviour between RRSO and 24 months |  |  |  |  |
| Not used | ·· | 39 (100%) | ·· | 39 (38%) |
| Stable | 46 (73%) | ·· | ·· | 46 (44%) |
| Increase | 11 (17%) | ·· | ·· | 11 (11%) |
| Decrease | 3 (5%) | ·· | ·· | 3 (3%) |
| Variable during the same period (both increase and decrease) | 3 (5%) | ·· | ·· | 3 (3%) |
| Unknown | ·· | ·· | 2 (100%) | 2 (2%) |
| Study period started vaginal estrogen |  |  |  |  |
| Not used | 58 (92%) | 36 (92%) | 1 (50%) | 95 (91%) |
| Between RRSO and 3 months | 1 (2%) | 1 (3%) | 0 (0%) | 2 (2%) |
| Between 3 months and 6 months after RRSO | 0 (0%) | 1 (3%) | 0 (0%) | 1 (1%) |
| Between 6 months and 12 months after RRSO | 1 (2%) | 0 (0%) | 0 (0%) | 1 (1%) |
| Between 12 months and 24 months after RRSO | 3 (5%) | 1 (3%) | 0 (0%) | 4 (4%) |
| Unknown | 0 (0%) | 0 (0%) | 1 (50%) | 1 (1%) |
| Study period started systemic testosterone (transdermal) |  |  |  |  |
| Not used | 59 (94%) | 39 (100%) | 0 (0%) | 98 (94%) |
| Between 3 months and 6 months after RRSO | 1 (2%) | 0 (0%) | 0 (0%) | 1 (1%) |
| Between 6 months and 12 months after RRSO | 2 (3%) | 0 (0%) | 0 (0%) | 2 (2%) |
| Between 12 months and 24 months after RRSO | 1 (2%) | 0 (0%) | 0 (0%) | 1 (1%) |
| Unknown | 0 (0%) | 0 (0%) | 2 (100%) | 2 (2%) |
| Data are presented as median (IQR) for continuous measures or n (%) for categorical measures. | | | | |
| IQR=Interquartile Range (25^th^ to 75^th^ percentile); IUD=Intrauterine Device; HRT= Hormone Replacement Therapy; RRSO=Risk-Reducing Salpingo-Oophorectomy; ·· = Not Applicable. | | | | |
| ^a^ "Other" estrogen users were those who switched between using oral, transdermal and/or subdermal estrogen formulations. | | | | |
| ^b^ Reasons for unknown HRT formulation: Withdrew or lost-to-follow-up prior to 24-month visit (n=12); Used Combination HRT and the formulation was unknown (n=1). | | | | |
| ^c^ Estrogen doses from different formulations were standardised according to the following equation: 50 mcg trandermal estradiol = 1 mg oral estradiol = 0.625 mg oral conjugated equine estrogen = 1 mg estradiol gel | | | | |
| ^d^ Adherence was defined as follows: (total number of days that HRT was used between surgery and 24 months or early discontinuation [whichever came first]) / (total number of days between surgery and 24 months or early discontinuation [whichever came first]) | | | | |
