# Supplementary material for: Sexual Function, Activity and Distress 24 Months After Surgical Menopause: What Happens After Menopause (WHAM)—A Prospective Controlled Study
Source: BJOG. 2026 Jan 22;133(6):1188–99. doi: 10.1111/1471-0528.70158 (PMC13040429; doi:10.1111/1471-0528.70158)
Supplement: Supplementary file 2 — Table S2: Descriptive statistics of Female Sexual Function Index (FSFI) in sexually active participants by timepoint and study group. [file BJO-133-1188-s006.docx]

**S2. Descriptive statistics of Female Sexual Function Index (FSFI) in sexually active participants by timepoint and study group.**

|  | **Baseline** | | **3 months** | | **6 months** | | **12 months** | | **24 months** | |
| --- | --- | --- | --- | --- | --- | --- | --- | --- | --- | --- |
|  | **RRSO** | **Comparison** | **RRSO** | **Comparison** | **RRSO** | **Comparison** | **RRSO** | **Comparison** | **RRSO** | **Comparison** |
|  | **N=77** | **N=80** | **N=73** | **N=80** | **N=74** | **N=78** | **N=67** | **N=79** | **N=58** | **N=68** |
| **1) Are you sexually active?** | | | | | | | | | | |
| No | 2 (3%) | 0 (0%) | 1 (1%) | 0 (0%) | 0 (0%) | 1 (1%) | 1 (1%) | 0 (0%) | 1 (2%) | 0 (0%) |
| Yes | 65 (84%) | 80 (100%) | 63 (86%) | 80 (100%) | 67 (91%) | 77 (99%) | 60 (90%) | 79 (100%) | 52 (90%) | 68 (100%) |
| Missing | 10 (13%) | 0 (0%) | 9 (12%) | 0 (0%) | 7 (9%) | 0 (0%) | 6 (9%) | 0 (0%) | 5 (9%) | 0 (0%) |
| **2) Do you have a sexual partner?** | | | | | | | | | | |
| No | 3 (4%) | 4 (5%) | 0 (0%) | 5 (6%) | 3 (4%) | 5 (6%) | 1 (1%) | 3 (4%) | 1 (2%) | 3 (4%) |
| Yes | 64 (83%) | 76 (95%) | 64 (88%) | 75 (94%) | 64 (86%) | 73 (94%) | 60 (90%) | 76 (96%) | 52 (90%) | 65 (96%) |
| Missing | 10 (13%) | 0 (0%) | 9 (12%) | 0 (0%) | 7 (9%) | 0 (0%) | 6 (9%) | 0 (0%) | 5 (9%) | 0 (0%) |
| **3) Do you have negative feelings towards your partner that impact on your sexual interest and/or activity?** | | | | | | | | | | |
| No | 59 (77%) | 60 (75%) | 60 (82%) | 61 (76%) | 57 (77%) | 58 (74%) | 54 (81%) | 66 (84%) | 48 (83%) | 52 (76%) |
| Yes | 5 (6%) | 16 (20%) | 4 (5%) | 14 (18%) | 7 (9%) | 14 (18%) | 6 (9%) | 10 (13%) | 4 (7%) | 13 (19%) |
| Missing | 13 (17%) | 4 (5%) | 9 (12%) | 5 (6%) | 10 (14%) | 6 (8%) | 7 (10%) | 3 (4%) | 6 (10%) | 3 (4%) |
| **4) Does your partner have sexual function problems that affect your sexual interest and/or activity?** | | | | | | | | | | |
| No | 58 (75%) | 68 (85%) | 60 (82%) | 71 (89%) | 61 (82%) | 67 (86%) | 55 (82%) | 70 (89%) | 49 (84%) | 59 (87%) |
| Yes | 6 (8%) | 8 (10%) | 4 (5%) | 4 (5%) | 3 (4%) | 5 (6%) | 5 (7%) | 6 (8%) | 3 (5%) | 6 (9%) |
| Missing | 13 (17%) | 4 (5%) | 9 (12%) | 5 (6%) | 10 (14%) | 6 (8%) | 7 (10%) | 3 (4%) | 6 (10%) | 3 (4%) |
| **5) Over the last 4 weeks, how often did you feel sexual desire or interest? *** | | | | | | | | | | |
| Almost never or never | 9 (9%) | 8 (8%) | 16 (15%) | 8 (8%) | 20 (19%) | 9 (9%) | 19 (18%) | 10 (10%) | 19 (18%) | 16 (16%) |
| A few times | 34 (33%) | 26 (25%) | 35 (34%) | 31 (30%) | 23 (22%) | 37 (36%) | 29 (28%) | 31 (30%) | 25 (24%) | 26 (25%) |
| Some of the time | 35 (34%) | 55 (54%) | 26 (25%) | 47 (46%) | 37 (36%) | 42 (41%) | 28 (27%) | 45 (44%) | 25 (24%) | 33 (32%) |
| Most of the time | 16 (15%) | 12 (12%) | 14 (13%) | 15 (15%) | 6 (6%) | 12 (12%) | 10 (10%) | 10 (10%) | 5 (5%) | 15 (15%) |
| Almost always or always | 6 (6%) | 1 (1%) | 4 (4%) | 1 (1%) | 4 (4%) | 1 (1%) | 3 (3%) | 1 (1%) | 4 (4%) | 2 (2%) |
| Missing | 4 (4%) | 0 (0%) | 9 (9%) | 0 (0%) | 14 (13%) | 1 (1%) | 15 (14%) | 5 (5%) | 26 (25%) | 10 (10%) |
| **6) Over the last 4 weeks, how much sexual desire or interest have you felt? *** | | | | | | | | | | |
| Very low or none at all | 11 (11%) | 9 (9%) | 16 (15%) | 9 (9%) | 20 (19%) | 10 (10%) | 19 (18%) | 8 (8%) | 19 (18%) | 18 (18%) |
| Low | 25 (24%) | 31 (30%) | 31 (30%) | 25 (25%) | 18 (17%) | 31 (30%) | 20 (19%) | 25 (25%) | 25 (24%) | 24 (24%) |
| Moderate | 45 (43%) | 47 (46%) | 33 (32%) | 52 (51%) | 38 (37%) | 48 (47%) | 40 (38%) | 50 (49%) | 27 (26%) | 33 (32%) |
| High | 13 (12%) | 14 (14%) | 12 (12%) | 15 (15%) | 13 (12%) | 12 (12%) | 8 (8%) | 12 (12%) | 5 (5%) | 15 (15%) |
| Very high | 7 (7%) | 1 (1%) | 3 (3%) | 1 (1%) | 1 (1%) | 0 (0%) | 1 (1%) | 2 (2%) | 2 (2%) | 2 (2%) |
| Missing | 3 (3%) | 0 (0%) | 9 (9%) | 0 (0%) | 14 (13%) | 1 (1%) | 16 (15%) | 5 (5%) | 26 (25%) | 10 (10%) |
| **7) Over the last 4 weeks, how often did you feel sexually aroused ("turned on") during sexual activity or intercourse?** | | | | | | | | | | |
| No sexual activity | 0 (0%) | 0 (0%) | 0 (0%) | 0 (0%) | 0 (0%) | 0 (0%) | 0 (0%) | 0 (0%) | 0 (0%) | 0 (0%) |
| Almost never or never | 1 (1%) | 3 (4%) | 5 (7%) | 0 (0%) | 6 (8%) | 0 (0%) | 2 (3%) | 1 (1%) | 2 (3%) | 1 (1%) |
| A few times | 9 (12%) | 9 (11%) | 13 (18%) | 6 (8%) | 11 (15%) | 9 (12%) | 17 (25%) | 7 (9%) | 12 (21%) | 7 (10%) |
| Sometimes | 8 (10%) | 9 (11%) | 6 (8%) | 11 (14%) | 12 (16%) | 6 (8%) | 7 (10%) | 9 (11%) | 11 (19%) | 11 (16%) |
| Most times | 30 (39%) | 17 (21%) | 21 (29%) | 25 (31%) | 23 (31%) | 19 (24%) | 13 (19%) | 17 (22%) | 17 (29%) | 14 (21%) |
| Almost always or always | 29 (38%) | 42 (52%) | 27 (37%) | 38 (48%) | 22 (30%) | 44 (56%) | 28 (42%) | 45 (57%) | 16 (28%) | 35 (51%) |
| Missing | 0 (0%) | 0 (0%) | 1 (1%) | 0 (0%) | 0 (0%) | 0 (0%) | 0 (0%) | 0 (0%) | 0 (0%) | 0 (0%) |
| **8) Over the last 4 weeks, how much sexual arousal ("turn on") have you felt during sexual activity or intercourse?** | | | | | | | | | | |
| No sexual activity | 0 (0%) | 0 (0%) | 0 (0%) | 0 (0%) | 0 (0%) | 0 (0%) | 0 (0%) | 0 (0%) | 0 (0%) | 0 (0%) |
| Very low or none at all | 0 (0%) | 1 (1%) | 4 (5%) | 0 (0%) | 5 (7%) | 0 (0%) | 3 (4%) | 1 (1%) | 1 (2%) | 2 (3%) |
| Low | 8 (10%) | 8 (10%) | 6 (8%) | 3 (4%) | 7 (9%) | 2 (3%) | 8 (12%) | 4 (5%) | 10 (17%) | 3 (4%) |
| Moderate | 22 (29%) | 24 (30%) | 24 (33%) | 21 (26%) | 23 (31%) | 24 (31%) | 23 (34%) | 19 (24%) | 22 (38%) | 20 (29%) |
| High | 29 (38%) | 30 (38%) | 23 (32%) | 33 (41%) | 26 (35%) | 27 (35%) | 21 (31%) | 26 (33%) | 16 (28%) | 23 (34%) |
| Very high | 18 (23%) | 17 (21%) | 15 (21%) | 23 (29%) | 13 (18%) | 25 (32%) | 12 (18%) | 29 (37%) | 9 (16%) | 20 (29%) |
| Missing | 0 (0%) | 0 (0%) | 1 (1%) | 0 (0%) | 0 (0%) | 0 (0%) | 0 (0%) | 0 (0%) | 0 (0%) | 0 (0%) |
| **9) Over the last 4 weeks, how confident were you about becoming sexually aroused during sexual activity or intercourse?** | | | | | | | | | | |
| No sexual activity | 0 (0%) | 0 (0%) | 0 (0%) | 0 (0%) | 0 (0%) | 0 (0%) | 0 (0%) | 0 (0%) | 0 (0%) | 0 (0%) |
| Very low or no confidence | 1 (1%) | 1 (1%) | 5 (7%) | 0 (0%) | 4 (5%) | 0 (0%) | 1 (1%) | 1 (1%) | 2 (3%) | 1 (1%) |
| Low confidence | 6 (8%) | 4 (5%) | 12 (16%) | 4 (5%) | 8 (11%) | 4 (5%) | 12 (18%) | 4 (5%) | 10 (17%) | 3 (4%) |
| Moderate confidence | 20 (26%) | 23 (29%) | 26 (36%) | 18 (22%) | 26 (35%) | 19 (24%) | 22 (33%) | 20 (25%) | 20 (34%) | 19 (28%) |
| High confidence | 25 (32%) | 24 (30%) | 12 (16%) | 25 (31%) | 22 (30%) | 23 (29%) | 17 (25%) | 21 (27%) | 19 (33%) | 17 (25%) |
| Very high confidence | 25 (32%) | 28 (35%) | 18 (25%) | 33 (41%) | 14 (19%) | 32 (41%) | 15 (22%) | 33 (42%) | 7 (12%) | 28 (41%) |
| Missing | 0 (0%) | 0 (0%) | 0 (0%) | 0 (0%) | 0 (0%) | 0 (0%) | 0 (0%) | 0 (0%) | 0 (0%) | 0 (0%) |
| **10) Over the last 4 weeks, how often have you been satisfied with your arousal (excitement) during sexual activity or intercourse?** | | | | | | | | | | |
| No sexual activity | 0 (0%) | 0 (0%) | 0 (0%) | 0 (0%) | 0 (0%) | 0 (0%) | 0 (0%) | 0 (0%) | 0 (0%) | 0 (0%) |
| Almost never or never | 3 (4%) | 5 (6%) | 5 (7%) | 0 (0%) | 7 (9%) | 2 (3%) | 2 (3%) | 1 (1%) | 5 (9%) | 1 (1%) |
| A few times | 2 (3%) | 4 (5%) | 8 (11%) | 5 (6%) | 7 (9%) | 4 (5%) | 12 (18%) | 7 (9%) | 8 (14%) | 4 (6%) |
| Sometimes | 8 (10%) | 6 (8%) | 8 (11%) | 3 (4%) | 9 (12%) | 5 (6%) | 9 (13%) | 6 (8%) | 6 (10%) | 7 (10%) |
| Most times | 30 (39%) | 30 (38%) | 26 (36%) | 34 (42%) | 29 (39%) | 24 (31%) | 20 (30%) | 19 (24%) | 19 (33%) | 19 (28%) |
| Almost always or always | 34 (44%) | 35 (44%) | 26 (36%) | 38 (48%) | 22 (30%) | 43 (55%) | 24 (36%) | 46 (58%) | 20 (34%) | 37 (54%) |
| Missing | 0 (0%) | 0 (0%) | 0 (0%) | 0 (0%) | 0 (0%) | 0 (0%) | 0 (0%) | 0 (0%) | 0 (0%) | 0 (0%) |
| **11) Over the last 4 weeks, how often did you become lubricated ("wet") during sexual activity or intercourse?** | | | | | | | | | | |
| No sexual activity | 0 (0%) | 0 (0%) | 0 (0%) | 0 (0%) | 0 (0%) | 0 (0%) | 0 (0%) | 0 (0%) | 0 (0%) | 0 (0%) |
| Almost never or never | 1 (1%) | 5 (6%) | 7 (10%) | 1 (1%) | 6 (8%) | 1 (1%) | 6 (9%) | 3 (4%) | 3 (5%) | 1 (1%) |
| A few times | 5 (6%) | 3 (4%) | 9 (12%) | 4 (5%) | 11 (15%) | 4 (5%) | 9 (13%) | 3 (4%) | 6 (10%) | 3 (4%) |
| Sometimes | 6 (8%) | 4 (5%) | 12 (16%) | 5 (6%) | 8 (11%) | 3 (4%) | 6 (9%) | 4 (5%) | 7 (12%) | 9 (13%) |
| Most times | 11 (14%) | 17 (21%) | 12 (16%) | 22 (28%) | 18 (24%) | 21 (27%) | 22 (33%) | 17 (22%) | 21 (36%) | 12 (18%) |
| Almost always or always | 54 (70%) | 51 (64%) | 33 (45%) | 48 (60%) | 31 (42%) | 49 (63%) | 24 (36%) | 52 (66%) | 20 (34%) | 43 (63%) |
| Missing | 0 (0%) | 0 (0%) | 0 (0%) | 0 (0%) | 0 (0%) | 0 (0%) | 0 (0%) | 0 (0%) | 1 (2%) | 0 (0%) |
| **12) Over the last 4 weeks, how difficult was it to become lubricated ("wet") during sexual activity or intercourse?** | | | | | | | | | | |
| No sexual activity | 0 (0%) | 0 (0%) | 0 (0%) | 0 (0%) | 0 (0%) | 0 (0%) | 0 (0%) | 0 (0%) | 0 (0%) | 0 (0%) |
| Extremely difficult or impossible | 1 (1%) | 1 (1%) | 0 (0%) | 0 (0%) | 3 (4%) | 0 (0%) | 4 (6%) | 1 (1%) | 3 (5%) | 1 (1%) |
| Very difficult | 0 (0%) | 3 (4%) | 5 (7%) | 0 (0%) | 4 (5%) | 1 (1%) | 2 (3%) | 1 (1%) | 3 (5%) | 0 (0%) |
| Difficult | 6 (8%) | 2 (2%) | 6 (8%) | 2 (2%) | 8 (11%) | 2 (3%) | 7 (10%) | 2 (3%) | 5 (9%) | 3 (4%) |
| Slightly difficult | 15 (19%) | 25 (31%) | 26 (36%) | 25 (31%) | 26 (35%) | 20 (26%) | 25 (37%) | 23 (29%) | 22 (38%) | 22 (32%) |
| Not difficult | 55 (71%) | 49 (61%) | 36 (49%) | 53 (66%) | 33 (45%) | 55 (71%) | 29 (43%) | 52 (66%) | 25 (43%) | 42 (62%) |
| Missing | 0 (0%) | 0 (0%) | 0 (0%) | 0 (0%) | 0 (0%) | 0 (0%) | 0 (0%) | 0 (0%) | 0 (0%) | 0 (0%) |
| **13) Over the last 4 weeks, how often did you maintain your lubrication ("wetness") until completion of sexual activity or intercourse?** | | | | | | | | | | |
| No sexual activity | 0 (0%) | 0 (0%) | 0 (0%) | 0 (0%) | 0 (0%) | 0 (0%) | 0 (0%) | 0 (0%) | 0 (0%) | 0 (0%) |
| Almost never or never | 1 (1%) | 4 (5%) | 6 (8%) | 2 (2%) | 9 (12%) | 1 (1%) | 6 (9%) | 3 (4%) | 5 (9%) | 1 (1%) |
| A few times | 4 (5%) | 5 (6%) | 11 (15%) | 6 (8%) | 7 (9%) | 2 (3%) | 4 (6%) | 4 (5%) | 1 (2%) | 6 (9%) |
| Sometimes | 2 (3%) | 4 (5%) | 7 (10%) | 9 (11%) | 6 (8%) | 6 (8%) | 8 (12%) | 4 (5%) | 7 (12%) | 6 (9%) |
| Most times | 16 (21%) | 17 (21%) | 18 (25%) | 14 (18%) | 22 (30%) | 16 (21%) | 12 (18%) | 16 (20%) | 20 (34%) | 12 (18%) |
| Almost always or always | 54 (70%) | 50 (62%) | 31 (42%) | 49 (61%) | 30 (41%) | 53 (68%) | 37 (55%) | 52 (66%) | 25 (43%) | 43 (63%) |
| Missing | 0 (0%) | 0 (0%) | 0 (0%) | 0 (0%) | 0 (0%) | 0 (0%) | 0 (0%) | 0 (0%) | 0 (0%) | 0 (0%) |
| **14) Over the last 4 weeks, how difficult was it to maintain your lubrication ("wetness") until completion of sexual activity or intercourse?** | | | | | | | | | | |
| No sexual activity | 0 (0%) | 0 (0%) | 0 (0%) | 0 (0%) | 0 (0%) | 0 (0%) | 0 (0%) | 0 (0%) | 0 (0%) | 0 (0%) |
| Extremely difficult or impossible | 1 (1%) | 2 (2%) | 2 (3%) | 2 (2%) | 3 (4%) | 1 (1%) | 4 (6%) | 1 (1%) | 3 (5%) | 1 (1%) |
| Very difficult | 1 (1%) | 2 (2%) | 3 (4%) | 0 (0%) | 3 (4%) | 0 (0%) | 0 (0%) | 1 (1%) | 2 (3%) | 0 (0%) |
| Difficult | 4 (5%) | 3 (4%) | 4 (5%) | 1 (1%) | 6 (8%) | 3 (4%) | 7 (10%) | 2 (3%) | 5 (9%) | 1 (1%) |
| Slightly difficult | 12 (16%) | 12 (15%) | 29 (40%) | 20 (25%) | 24 (32%) | 13 (17%) | 17 (25%) | 18 (23%) | 17 (29%) | 16 (24%) |
| Not difficult | 59 (77%) | 61 (76%) | 35 (48%) | 57 (71%) | 38 (51%) | 61 (78%) | 39 (58%) | 57 (72%) | 31 (53%) | 50 (74%) |
| Missing | 0 (0%) | 0 (0%) | 0 (0%) | 0 (0%) | 0 (0%) | 0 (0%) | 0 (0%) | 0 (0%) | 0 (0%) | 0 (0%) |
| **15) Over the last 4 weeks, when you had sexual stimulation or intercourse, how often did you reach orgasm (climax)?** | | | | | | | | | | |
| No sexual activity | 0 (0%) | 0 (0%) | 0 (0%) | 0 (0%) | 0 (0%) | 0 (0%) | 0 (0%) | 0 (0%) | 0 (0%) | 0 (0%) |
| Almost never or never | 3 (4%) | 4 (5%) | 6 (8%) | 2 (2%) | 7 (9%) | 4 (5%) | 5 (7%) | 3 (4%) | 7 (12%) | 4 (6%) |
| A few times | 4 (5%) | 3 (4%) | 9 (12%) | 9 (11%) | 6 (8%) | 8 (10%) | 9 (13%) | 6 (8%) | 8 (14%) | 8 (12%) |
| Sometimes | 10 (13%) | 9 (11%) | 9 (12%) | 10 (12%) | 9 (12%) | 4 (5%) | 9 (13%) | 8 (10%) | 4 (7%) | 4 (6%) |
| Most times | 24 (31%) | 23 (29%) | 22 (30%) | 19 (24%) | 24 (32%) | 16 (21%) | 19 (28%) | 20 (25%) | 12 (21%) | 17 (25%) |
| Almost always or always | 36 (47%) | 41 (51%) | 27 (37%) | 40 (50%) | 28 (38%) | 46 (59%) | 25 (37%) | 42 (53%) | 27 (47%) | 35 (51%) |
| Missing | 0 (0%) | 0 (0%) | 0 (0%) | 0 (0%) | 0 (0%) | 0 (0%) | 0 (0%) | 0 (0%) | 0 (0%) | 0 (0%) |
| **16) Over the last 4 weeks, when you had sexual stimulation or intercourse, how difficult was it for you to reach orgasm ("climax")?** | | | | | | | | | | |
| No sexual activity | 0 (0%) | 0 (0%) | 0 (0%) | 0 (0%) | 0 (0%) | 0 (0%) | 0 (0%) | 0 (0%) | 0 (0%) | 0 (0%) |
| Extremely difficult or impossible | 3 (4%) | 3 (4%) | 2 (3%) | 1 (1%) | 6 (8%) | 1 (1%) | 4 (6%) | 1 (1%) | 4 (7%) | 3 (4%) |
| Very difficult | 0 (0%) | 1 (1%) | 6 (8%) | 0 (0%) | 2 (3%) | 0 (0%) | 3 (4%) | 2 (3%) | 5 (9%) | 2 (3%) |
| Difficult | 6 (8%) | 8 (10%) | 3 (4%) | 6 (8%) | 6 (8%) | 7 (9%) | 5 (7%) | 5 (6%) | 5 (9%) | 2 (3%) |
| Slightly difficult | 24 (31%) | 26 (32%) | 31 (42%) | 27 (34%) | 24 (32%) | 26 (33%) | 32 (48%) | 34 (43%) | 20 (34%) | 27 (40%) |
| Not difficult | 44 (57%) | 42 (52%) | 31 (42%) | 46 (57%) | 36 (49%) | 44 (56%) | 23 (34%) | 37 (47%) | 24 (41%) | 34 (50%) |
| Missing | 0 (0%) | 0 (0%) | 0 (0%) | 0 (0%) | 0 (0%) | 0 (0%) | 0 (0%) | 0 (0%) | 0 (0%) | 0 (0%) |
| **17) Over the last 4 weeks, how satisfied were you with your ability to reach orgasm ("climax") during sexual activity or intercourse?** | | | | | | | | | | |
| No sexual activity | 0 (0%) | 0 (0%) | 0 (0%) | 0 (0%) | 0 (0%) | 0 (0%) | 0 (0%) | 0 (0%) | 0 (0%) | 0 (0%) |
| Very dissatisfied | 3 (4%) | 3 (4%) | 5 (7%) | 2 (2%) | 5 (7%) | 1 (1%) | 4 (6%) | 2 (3%) | 3 (5%) | 2 (3%) |
| Moderately dissatisfied | 4 (5%) | 3 (4%) | 3 (4%) | 4 (5%) | 6 (8%) | 6 (8%) | 7 (10%) | 4 (5%) | 12 (21%) | 6 (9%) |
| About equally satisfied and dissatisfied | 4 (5%) | 10 (12%) | 10 (14%) | 7 (9%) | 12 (16%) | 6 (8%) | 7 (10%) | 9 (11%) | 7 (12%) | 9 (13%) |
| Moderately satisfied | 19 (25%) | 19 (24%) | 23 (32%) | 23 (29%) | 20 (27%) | 21 (27%) | 24 (36%) | 24 (30%) | 15 (26%) | 16 (24%) |
| Very satisfied | 47 (61%) | 45 (56%) | 32 (44%) | 44 (55%) | 31 (42%) | 44 (56%) | 25 (37%) | 40 (51%) | 21 (36%) | 35 (51%) |
| Missing | 0 (0%) | 0 (0%) | 0 (0%) | 0 (0%) | 0 (0%) | 0 (0%) | 0 (0%) | 0 (0%) | 0 (0%) | 0 (0%) |
| **18) Over the last 4 weeks, how satisfied have you been with the amount of emotional closeness during sexual activity between you and your partner?** | | | | | | | | | | |
| No sexual activity | 0 (0%) | 0 (0%) | 0 (0%) | 0 (0%) | 0 (0%) | 0 (0%) | 0 (0%) | 0 (0%) | 0 (0%) | 0 (0%) |
| Very dissatisfied | 2 (3%) | 4 (5%) | 2 (3%) | 1 (1%) | 4 (5%) | 3 (4%) | 3 (4%) | 5 (6%) | 3 (5%) | 1 (1%) |
| Moderately dissatisfied | 1 (1%) | 5 (6%) | 1 (1%) | 2 (2%) | 6 (8%) | 7 (9%) | 1 (1%) | 6 (8%) | 5 (9%) | 3 (4%) |
| About equally satisfied and dissatisfied | 8 (10%) | 9 (11%) | 10 (14%) | 12 (15%) | 5 (7%) | 2 (3%) | 11 (16%) | 5 (6%) | 5 (9%) | 7 (10%) |
| Moderately satisfied | 17 (22%) | 22 (28%) | 17 (23%) | 27 (34%) | 11 (15%) | 29 (37%) | 15 (22%) | 23 (29%) | 19 (33%) | 20 (29%) |
| Very satisfied | 47 (61%) | 36 (45%) | 43 (59%) | 33 (41%) | 44 (59%) | 32 (41%) | 36 (54%) | 37 (47%) | 25 (43%) | 34 (50%) |
| Missing | 2 (3%) | 4 (5%) | 0 (0%) | 5 (6%) | 4 (5%) | 5 (6%) | 1 (1%) | 3 (4%) | 1 (2%) | 3 (4%) |
| **19) Over the last 4 weeks, how satisfied have you been with your sexual relationship with your partner?** | | | | | | | | | | |
| Very dissatisfied | 2 (3%) | 2 (2%) | 3 (4%) | 1 (1%) | 6 (8%) | 3 (4%) | 6 (9%) | 4 (5%) | 3 (5%) | 1 (1%) |
| Moderately dissatisfied | 6 (8%) | 10 (12%) | 9 (12%) | 5 (6%) | 6 (8%) | 7 (9%) | 5 (7%) | 8 (10%) | 12 (21%) | 1 (1%) |
| About equally satisfied and dissatisfied | 5 (6%) | 13 (16%) | 9 (12%) | 15 (19%) | 10 (14%) | 14 (18%) | 9 (13%) | 9 (11%) | 10 (17%) | 11 (16%) |
| Moderately satisfied | 24 (31%) | 23 (29%) | 18 (25%) | 30 (38%) | 20 (27%) | 28 (36%) | 22 (33%) | 32 (41%) | 13 (22%) | 28 (41%) |
| Very satisfied | 38 (49%) | 28 (35%) | 34 (47%) | 24 (30%) | 28 (38%) | 21 (27%) | 24 (36%) | 23 (29%) | 19 (33%) | 24 (35%) |
| Missing | 2 (3%) | 4 (5%) | 0 (0%) | 5 (6%) | 4 (5%) | 5 (6%) | 1 (1%) | 3 (4%) | 1 (2%) | 3 (4%) |
| **20) Over the last 4 weeks, how satisfied have you been with your sex life overall?** | | | | | | | | | | |
| Very dissatisfied | 2 (3%) | 3 (4%) | 4 (5%) | 0 (0%) | 7 (9%) | 4 (5%) | 6 (9%) | 3 (4%) | 2 (3%) | 1 (1%) |
| Moderately dissatisfied | 9 (12%) | 10 (12%) | 13 (18%) | 7 (9%) | 6 (8%) | 7 (9%) | 5 (7%) | 7 (9%) | 12 (21%) | 3 (4%) |
| About equally satisfied and dissatisfied | 4 (5%) | 14 (18%) | 8 (11%) | 18 (22%) | 16 (22%) | 15 (19%) | 8 (12%) | 16 (20%) | 11 (19%) | 8 (12%) |
| Moderately satisfied | 32 (42%) | 29 (36%) | 22 (30%) | 31 (39%) | 21 (28%) | 33 (42%) | 27 (40%) | 36 (46%) | 20 (34%) | 34 (50%) |
| Very satisfied | 30 (39%) | 24 (30%) | 26 (36%) | 24 (30%) | 23 (31%) | 19 (24%) | 21 (31%) | 17 (22%) | 13 (22%) | 22 (32%) |
| Missing | 0 (0%) | 0 (0%) | 0 (0%) | 0 (0%) | 1 (1%) | 0 (0%) | 0 (0%) | 0 (0%) | 0 (0%) | 0 (0%) |
| **21) Over the last 4 weeks, how often did you experience discomfort or pain during vaginal penetration?** | | | | | | | | | | |
| Did not attempt intercourse | 0 (0%) | 0 (0%) | 0 (0%) | 0 (0%) | 0 (0%) | 0 (0%) | 0 (0%) | 0 (0%) | 0 (0%) | 0 (0%) |
| Almost always or always | 1 (1%) | 1 (1%) | 0 (0%) | 1 (1%) | 7 (9%) | 1 (1%) | 2 (3%) | 1 (1%) | 4 (7%) | 1 (1%) |
| Most times | 3 (4%) | 3 (4%) | 7 (10%) | 2 (2%) | 5 (7%) | 1 (1%) | 1 (1%) | 0 (0%) | 0 (0%) | 1 (1%) |
| Sometimes | 6 (8%) | 8 (10%) | 10 (14%) | 3 (4%) | 8 (11%) | 4 (5%) | 9 (13%) | 4 (5%) | 6 (10%) | 4 (6%) |
| A few times | 9 (12%) | 13 (16%) | 17 (23%) | 10 (12%) | 9 (12%) | 9 (12%) | 9 (13%) | 11 (14%) | 8 (14%) | 8 (12%) |
| Almost never or never | 58 (75%) | 55 (69%) | 39 (53%) | 64 (80%) | 45 (61%) | 63 (81%) | 46 (69%) | 63 (80%) | 40 (69%) | 54 (79%) |
| Missing | 0 (0%) | 0 (0%) | 0 (0%) | 0 (0%) | 0 (0%) | 0 (0%) | 0 (0%) | 0 (0%) | 0 (0%) | 0 (0%) |
| **22) Over the last 4 weeks, how often did you experience discomfort or pain following vaginal penetration?** | | | | | | | | | | |
| Did not attempt intercourse | 0 (0%) | 0 (0%) | 0 (0%) | 0 (0%) | 0 (0%) | 0 (0%) | 0 (0%) | 0 (0%) | 0 (0%) | 0 (0%) |
| Almost always or always | 1 (1%) | 0 (0%) | 0 (0%) | 0 (0%) | 2 (3%) | 1 (1%) | 2 (3%) | 1 (1%) | 1 (2%) | 0 (0%) |
| Most times | 2 (3%) | 0 (0%) | 2 (3%) | 1 (1%) | 3 (4%) | 1 (1%) | 1 (1%) | 0 (0%) | 4 (7%) | 0 (0%) |
| Sometimes | 4 (5%) | 5 (6%) | 7 (10%) | 0 (0%) | 6 (8%) | 1 (1%) | 6 (9%) | 1 (1%) | 3 (5%) | 2 (3%) |
| A few times | 5 (6%) | 8 (10%) | 13 (18%) | 9 (11%) | 8 (11%) | 6 (8%) | 8 (12%) | 11 (14%) | 2 (3%) | 10 (15%) |
| Almost never or never | 64 (83%) | 67 (84%) | 51 (70%) | 70 (88%) | 55 (74%) | 69 (88%) | 50 (75%) | 66 (84%) | 48 (83%) | 56 (82%) |
| Missing | 1 (1%) | 0 (0%) | 0 (0%) | 0 (0%) | 0 (0%) | 0 (0%) | 0 (0%) | 0 (0%) | 0 (0%) | 0 (0%) |
| **23) Over the last 4 weeks, how much discomfort or pain have you felt during or following vaginal penetration?** | | | | | | | | | | |
| Did not attempt intercourse | 0 (0%) | 0 (0%) | 0 (0%) | 0 (0%) | 0 (0%) | 0 (0%) | 0 (0%) | 0 (0%) | 0 (0%) | 0 (0%) |
| Very high | 0 (0%) | 0 (0%) | 0 (0%) | 0 (0%) | 0 (0%) | 0 (0%) | 0 (0%) | 0 (0%) | 1 (2%) | 0 (0%) |
| High | 1 (1%) | 1 (1%) | 2 (3%) | 0 (0%) | 2 (3%) | 1 (1%) | 3 (4%) | 0 (0%) | 4 (7%) | 0 (0%) |
| Moderate | 6 (8%) | 5 (6%) | 8 (11%) | 4 (5%) | 10 (14%) | 2 (3%) | 6 (9%) | 4 (5%) | 3 (5%) | 4 (6%) |
| Low | 12 (16%) | 17 (21%) | 17 (23%) | 17 (21%) | 12 (16%) | 12 (15%) | 8 (12%) | 17 (22%) | 9 (16%) | 11 (16%) |
| Very low or none at all | 58 (75%) | 57 (71%) | 46 (63%) | 59 (74%) | 50 (68%) | 63 (81%) | 50 (75%) | 58 (73%) | 41 (71%) | 53 (78%) |
| Missing | 0 (0%) | 0 (0%) | 0 (0%) | 0 (0%) | 0 (0%) | 0 (0%) | 0 (0%) | 0 (0%) | 0 (0%) | 0 (0%) |
| Data are presented as n/N (%) for categorical measures. RRSO= Risk-Reducing Salpingo-Oophorectomy.  * All participants with available data were included, regardless of being sexually active or not. | | | | | | | | | | |
