# Supplementary material for: Sexual Function, Activity and Distress 24 Months After Surgical Menopause: What Happens After Menopause (WHAM)—A Prospective Controlled Study
Source: BJOG. 2026 Jan 22;133(6):1188–99. doi: 10.1111/1471-0528.70158 (PMC13040429; doi:10.1111/1471-0528.70158)
Supplement: Supplementary file 3 — Table S3: Descriptive statistics of Sexual Activity Questionnaire (SAQ) in sexually active participants by timepoint and study group. [file BJO-133-1188-s004.docx]

**S3. Descriptive statistics of Sexual Activity Questionnaire (SAQ) in sexually active participants by timepoint and study group.**

|  | **Baseline** | | **3 months** | | **6 months** | | **12 months** | | **24 months** | |
| --- | --- | --- | --- | --- | --- | --- | --- | --- | --- | --- |
|  | **RRSO** | **Comparison** | **RRSO** | **Comparison** | **RRSO** | **Comparison** | **RRSO** | **Comparison** | **RRSO** | **Comparison** |
|  | **N=69** | **N=79** | **N=78** | **N=77** | **N=71** | **N=72** | **N=69** | **N=74** | **N=60** | **N=67** |
| **Are you currently married or having an intimate relationship with someone?** | | | | | | | | | | |
| No | 0 (0%) | 1 (1%) | 0 (0%) | 0 (0%) | 0 (0%) | 0 (0%) | 0 (0%) | 0 (0%) | 0 (0%) | 2 (3%) |
| Yes | 59 (86%) | 76 (96%) | 61 (78%) | 76 (99%) | 60 (85%) | 72 (100%) | 60 (87%) | 74 (100%) | 51 (85%) | 65 (97%) |
| Missing | 10 (14%) | 2 (3%) | 17 (22%) | 1 (1%) | 11 (15%) | 0 (0%) | 9 (13%) | 0 (0%) | 9 (15%) | 0 (0%) |
| **Have you changed your sexual partner in the last 6 months?** | | | | | | | | | | |
| No | 55 (80%) | 74 (94%) | 58 (74%) | 76 (99%) | 58 (82%) | 69 (96%) | 59 (86%) | 70 (95%) | 51 (85%) | 63 (94%) |
| Yes | 0 (0%) | 2 (3%) | 2 (3%) | 0 (0%) | 0 (0%) | 2 (3%) | 1 (1%) | 4 (5%) | 0 (0%) | 4 (6%) |
| Missing | 14 (20%) | 3 (4%) | 18 (23%) | 1 (1%) | 13 (18%) | 1 (1%) | 9 (13%) | 0 (0%) | 9 (15%) | 0 (0%) |
| **Do you engage in sexual activity with anyone at the moment?** | | | | | | | | | | |
| No | 0 (0%) | 0 (0%) | 0 (0%) | 0 (0%) | 0 (0%) | 0 (0%) | 0 (0%) | 0 (0%) | 0 (0%) | 0 (0%) |
| Yes | 59 (86%) | 77 (97%) | 61 (78%) | 76 (99%) | 60 (85%) | 72 (100%) | 60 (87%) | 74 (100%) | 51 (85%) | 67 (100%) |
| Missing | 10 (14%) | 2 (3%) | 17 (22%) | 1 (1%) | 11 (15%) | 0 (0%) | 9 (13%) | 0 (0%) | 9 (15%) | 0 (0%) |
| **1) Was having sex an important part of your life this month?** | | | | | | | | | | |
| Very much | 14 (20%) | 15 (19%) | 17 (22%) | 10 (13%) | 12 (17%) | 14 (19%) | 14 (20%) | 11 (15%) | 11 (18%) | 11 (16%) |
| Somewhat | 24 (35%) | 26 (33%) | 20 (26%) | 40 (52%) | 24 (34%) | 26 (36%) | 20 (29%) | 33 (45%) | 16 (27%) | 24 (36%) |
| A little | 20 (29%) | 22 (28%) | 20 (26%) | 20 (26%) | 26 (37%) | 21 (29%) | 20 (29%) | 18 (24%) | 23 (38%) | 20 (30%) |
| Not at all | 11 (16%) | 15 (19%) | 19 (24%) | 7 (9%) | 9 (13%) | 11 (15%) | 14 (20%) | 12 (16%) | 10 (17%) | 12 (18%) |
| Missing | 0 (0%) | 1 (1%) | 2 (3%) | 0 (0%) | 0 (0%) | 0 (0%) | 1 (1%) | 0 (0%) | 0 (0%) | 0 (0%) |
| **2) Did you enjoy sexual activity this month?** | | | | | | | | | | |
| Very much | 39 (57%) | 42 (53%) | 32 (41%) | 37 (48%) | 28 (39%) | 34 (47%) | 22 (32%) | 36 (49%) | 19 (32%) | 27 (40%) |
| Somewhat | 20 (29%) | 25 (32%) | 26 (33%) | 35 (45%) | 25 (35%) | 23 (32%) | 26 (38%) | 29 (39%) | 22 (37%) | 31 (46%) |
| A little | 7 (10%) | 9 (11%) | 14 (18%) | 5 (6%) | 12 (17%) | 14 (19%) | 17 (25%) | 8 (11%) | 14 (23%) | 6 (9%) |
| Not at all | 3 (4%) | 1 (1%) | 3 (4%) | 0 (0%) | 6 (8%) | 1 (1%) | 1 (1%) | 1 (1%) | 5 (8%) | 2 (3%) |
| Missing | 0 (0%) | 2 (3%) | 3 (4%) | 0 (0%) | 0 (0%) | 0 (0%) | 3 (4%) | 0 (0%) | 0 (0%) | 1 (1%) |
| **3) In general, were you too tired to have sex?** | | | | | | | | | | |
| Very much | 13 (19%) | 11 (14%) | 14 (18%) | 6 (8%) | 11 (15%) | 12 (17%) | 12 (17%) | 12 (16%) | 10 (17%) | 10 (15%) |
| Somewhat | 25 (36%) | 32 (41%) | 18 (23%) | 26 (34%) | 21 (30%) | 23 (32%) | 19 (28%) | 22 (30%) | 21 (35%) | 26 (39%) |
| A little | 15 (22%) | 26 (33%) | 27 (35%) | 34 (44%) | 24 (34%) | 28 (39%) | 24 (35%) | 28 (38%) | 18 (30%) | 19 (28%) |
| Not at all | 16 (23%) | 10 (13%) | 17 (22%) | 11 (14%) | 15 (21%) | 9 (12%) | 12 (17%) | 12 (16%) | 10 (17%) | 11 (16%) |
| Missing | 0 (0%) | 0 (0%) | 2 (3%) | 0 (0%) | 0 (0%) | 0 (0%) | 2 (3%) | 0 (0%) | 1 (2%) | 1 (1%) |
| **4) Did you desire to have sex with your partner(s) this month?** | | | | | | | | | | |
| Very much | 29 (42%) | 28 (35%) | 25 (32%) | 30 (39%) | 21 (30%) | 23 (32%) | 16 (23%) | 28 (38%) | 11 (18%) | 17 (25%) |
| Somewhat | 22 (32%) | 26 (33%) | 22 (28%) | 33 (43%) | 23 (32%) | 24 (33%) | 30 (43%) | 27 (36%) | 26 (43%) | 26 (39%) |
| A little | 15 (22%) | 18 (23%) | 19 (24%) | 14 (18%) | 19 (27%) | 22 (31%) | 12 (17%) | 16 (22%) | 19 (32%) | 18 (27%) |
| Not at all | 3 (4%) | 7 (9%) | 9 (12%) | 0 (0%) | 7 (10%) | 3 (4%) | 8 (12%) | 3 (4%) | 4 (7%) | 6 (9%) |
| Missing | 0 (0%) | 0 (0%) | 3 (4%) | 0 (0%) | 1 (1%) | 0 (0%) | 3 (4%) | 0 (0%) | 0 (0%) | 0 (0%) |
| **5) During sexual relations, how frequently did you notice dryness of your vagina this month?** | | | | | | | | | | |
| Very much | 2 (3%) | 3 (4%) | 10 (13%) | 2 (3%) | 10 (14%) | 3 (4%) | 6 (9%) | 2 (3%) | 9 (15%) | 2 (3%) |
| Somewhat | 6 (9%) | 9 (11%) | 15 (19%) | 4 (5%) | 9 (13%) | 3 (4%) | 11 (16%) | 7 (9%) | 10 (17%) | 2 (3%) |
| A little | 17 (25%) | 25 (32%) | 17 (22%) | 25 (32%) | 21 (30%) | 20 (28%) | 18 (26%) | 16 (22%) | 18 (30%) | 25 (37%) |
| Not at all | 44 (64%) | 40 (51%) | 30 (38%) | 46 (60%) | 29 (41%) | 46 (64%) | 31 (45%) | 48 (65%) | 22 (37%) | 37 (55%) |
| Missing | 0 (0%) | 2 (3%) | 6 (8%) | 0 (0%) | 2 (3%) | 0 (0%) | 3 (4%) | 1 (1%) | 1 (2%) | 1 (1%) |
| **6) Did you feel pain or discomfort during penetration this month?** | | | | | | | | | | |
| Very much | 1 (1%) | 1 (1%) | 2 (3%) | 1 (1%) | 2 (3%) | 0 (0%) | 2 (3%) | 1 (1%) | 4 (7%) | 0 (0%) |
| Somewhat | 3 (4%) | 5 (6%) | 9 (12%) | 2 (3%) | 7 (10%) | 4 (6%) | 6 (9%) | 3 (4%) | 5 (8%) | 3 (4%) |
| A little | 10 (14%) | 17 (22%) | 23 (29%) | 17 (22%) | 19 (27%) | 12 (17%) | 10 (14%) | 14 (19%) | 11 (18%) | 12 (18%) |
| Not at all | 53 (77%) | 51 (65%) | 38 (49%) | 56 (73%) | 38 (54%) | 56 (78%) | 47 (68%) | 54 (73%) | 37 (62%) | 51 (76%) |
| Missing | 2 (3%) | 5 (6%) | 6 (8%) | 1 (1%) | 5 (7%) | 0 (0%) | 4 (6%) | 2 (3%) | 3 (5%) | 1 (1%) |
| **7) Have you experienced bleeding during intercourse?** | | | | | | | | | | |
| Very much | 0 (0%) | 0 (0%) | 1 (1%) | 0 (0%) | 1 (1%) | 1 (1%) | 1 (1%) | 1 (1%) | 1 (2%) | 0 (0%) |
| Somewhat | 1 (1%) | 0 (0%) | 5 (6%) | 3 (4%) | 1 (1%) | 1 (1%) | 0 (0%) | 0 (0%) | 0 (0%) | 1 (1%) |
| A little | 3 (4%) | 5 (6%) | 10 (13%) | 5 (6%) | 6 (8%) | 2 (3%) | 7 (10%) | 2 (3%) | 0 (0%) | 3 (4%) |
| Not at all | 63 (91%) | 70 (89%) | 46 (59%) | 68 (88%) | 51 (72%) | 67 (93%) | 51 (74%) | 70 (95%) | 47 (78%) | 62 (93%) |
| Missing | 2 (3%) | 4 (5%) | 16 (21%) | 1 (1%) | 12 (17%) | 1 (1%) | 10 (14%) | 1 (1%) | 12 (20%) | 1 (1%) |
| **7a) If yes, has it bothered you? ^a^** | | | | | | | | | | |
| Very much | 0 (0%) | 0 (0%) | 2 (12%) | 0 (0%) | 2 (25%) | 0 (0%) | 2 (25%) | 0 (0%) | 1 (100%) | 0 (0%) |
| Somewhat | 0 (0%) | 1 (20%) | 1 (6%) | 1 (12%) | 1 (12%) | 2 (50%) | 1 (12%) | 0 (0%) | 0 (0%) | 0 (0%) |
| A little | 2 (50%) | 0 (0%) | 5 (31%) | 2 (25%) | 2 (25%) | 0 (0%) | 1 (12%) | 2 (67%) | 0 (0%) | 2 (50%) |
| Not at all | 2 (50%) | 4 (80%) | 7 (44%) | 5 (62%) | 3 (38%) | 1 (25%) | 4 (50%) | 1 (33%) | 0 (0%) | 2 (50%) |
| Missing | 0 (0%) | 0 (0%) | 1 (6%) | 0 (0%) | 0 (0%) | 1 (25%) | 0 (0%) | 0 (0%) | 0 (0%) | 0 (0%) |
| **8) In general, did you feel satisfied after sexual activity this month?** | | | | | | | | | | |
| Very much | 37 (54%) | 41 (52%) | 27 (35%) | 37 (48%) | 21 (30%) | 41 (57%) | 26 (38%) | 41 (55%) | 18 (30%) | 34 (51%) |
| Somewhat | 22 (32%) | 26 (33%) | 31 (40%) | 30 (39%) | 30 (42%) | 21 (29%) | 25 (36%) | 22 (30%) | 22 (37%) | 25 (37%) |
| A little | 7 (10%) | 9 (11%) | 13 (17%) | 9 (12%) | 16 (23%) | 8 (11%) | 13 (19%) | 9 (12%) | 15 (25%) | 5 (7%) |
| Not at all | 2 (3%) | 1 (1%) | 3 (4%) | 1 (1%) | 4 (6%) | 2 (3%) | 2 (3%) | 1 (1%) | 3 (5%) | 2 (3%) |
| Missing | 1 (1%) | 2 (3%) | 4 (5%) | 0 (0%) | 0 (0%) | 0 (0%) | 3 (4%) | 1 (1%) | 2 (3%) | 1 (1%) |
| **9) How often did you engage in sexual activity this month?** | | | | | | | | | | |
| 5 times or more | 24 (35%) | 20 (25%) | 26 (33%) | 27 (35%) | 24 (34%) | 26 (36%) | 16 (23%) | 20 (27%) | 17 (28%) | 19 (28%) |
| 3-4 times | 23 (33%) | 27 (34%) | 22 (28%) | 33 (43%) | 27 (38%) | 19 (26%) | 27 (39%) | 29 (39%) | 22 (37%) | 25 (37%) |
| 1-2 times | 19 (28%) | 29 (37%) | 27 (35%) | 17 (22%) | 18 (25%) | 27 (38%) | 23 (33%) | 24 (32%) | 20 (33%) | 22 (33%) |
| Not at all | 3 (4%) | 3 (4%) | 2 (3%) | 0 (0%) | 2 (3%) | 0 (0%) | 2 (3%) | 1 (1%) | 1 (2%) | 1 (1%) |
| Missing | 0 (0%) | 0 (0%) | 1 (1%) | 0 (0%) | 0 (0%) | 0 (0%) | 1 (1%) | 0 (0%) | 0 (0%) | 0 (0%) |
| **10) How did this frequency of sexual activity compare with what is usual for you?** | | | | | | | | | | |
| Much more | 5 (7%) | 2 (3%) | 6 (8%) | 2 (3%) | 2 (3%) | 1 (1%) | 3 (4%) | 5 (7%) | 0 (0%) | 0 (0%) |
| Somewhat more | 10 (14%) | 5 (6%) | 10 (13%) | 14 (18%) | 9 (13%) | 8 (11%) | 10 (14%) | 5 (7%) | 11 (18%) | 12 (18%) |
| About the same | 43 (62%) | 54 (68%) | 34 (44%) | 49 (64%) | 37 (52%) | 46 (64%) | 31 (45%) | 44 (59%) | 32 (53%) | 35 (52%) |
| Less than usual | 11 (16%) | 18 (23%) | 27 (35%) | 12 (16%) | 21 (30%) | 17 (24%) | 24 (35%) | 20 (27%) | 17 (28%) | 20 (30%) |
| Missing | 0 (0%) | 0 (0%) | 1 (1%) | 0 (0%) | 2 (3%) | 0 (0%) | 1 (1%) | 0 (0%) | 0 (0%) | 0 (0%) |
| **11) Were you satisfied with the frequency of sexual activity this month?** | | | | | | | | | | |
| Very much | 26 (38%) | 25 (32%) | 28 (36%) | 28 (36%) | 27 (38%) | 22 (31%) | 23 (33%) | 20 (27%) | 20 (33%) | 20 (30%) |
| Somewhat | 26 (38%) | 37 (47%) | 23 (29%) | 38 (49%) | 21 (30%) | 32 (44%) | 22 (32%) | 33 (45%) | 21 (35%) | 32 (48%) |
| A little | 8 (12%) | 15 (19%) | 15 (19%) | 9 (12%) | 15 (21%) | 13 (18%) | 13 (19%) | 16 (22%) | 12 (20%) | 12 (18%) |
| Not at all | 9 (13%) | 2 (3%) | 11 (14%) | 2 (3%) | 8 (11%) | 5 (7%) | 9 (13%) | 5 (7%) | 7 (12%) | 3 (4%) |
| Missing | 0 (0%) | 0 (0%) | 1 (1%) | 0 (0%) | 0 (0%) | 0 (0%) | 2 (3%) | 0 (0%) | 0 (0%) | 0 (0%) |
| Data are presented as n/N (%) for categorical measures. RRSO= Risk-Reducing Salpingo-Oophorectomy. | | | | | | | | | | |
| ^a^ Participants who did not answer 'Not at all' or 'Missing' in '7) Have you experienced bleeding during intercourse?' were included as denominators | | | | | | | | | | |
