# Supplementary material for: Sexual Function, Activity and Distress 24 Months After Surgical Menopause: What Happens After Menopause (WHAM)—A Prospective Controlled Study
Source: BJOG. 2026 Jan 22;133(6):1188–99. doi: 10.1111/1471-0528.70158 (PMC13040429; doi:10.1111/1471-0528.70158)
Supplement: Supplementary file 4 — Table S4: Descriptive statistics of Revised Female Sexual Distress Scale in all participants by timepoint and study group. [file BJO-133-1188-s005.docx]

**S4. Descriptive statistics of Revised Female Sexual Distress Scale in all participants by timepoint and study group.**

|  | **Baseline** | | **3 months** | | **6 months** | | **12 months** | | **24 months** | |
| --- | --- | --- | --- | --- | --- | --- | --- | --- | --- | --- |
|  | **RRSO** | **Comparison** | **RRSO** | **Comparison** | **RRSO** | **Comparison** | **RRSO** | **Comparison** | **RRSO** | **Comparison** |
|  | **N=104** | **N=102** | **N=104** | **N=102** | **N=104** | **N=102** | **N=104** | **N=102** | **N=104** | **N=102** |
| **1) Distressed about your sex life** | | | | | | | | | | |
| Never | 42 (40%) | 42 (41%) | 39 (38%) | 49 (48%) | 32 (31%) | 46 (45%) | 36 (35%) | 47 (46%) | 30 (29%) | 48 (47%) |
| Rarely | 19 (18%) | 32 (31%) | 26 (25%) | 30 (29%) | 24 (23%) | 32 (31%) | 24 (23%) | 30 (29%) | 18 (17%) | 28 (27%) |
| Occasionally | 19 (18%) | 27 (26%) | 24 (23%) | 20 (20%) | 24 (23%) | 20 (20%) | 19 (18%) | 18 (18%) | 22 (21%) | 11 (11%) |
| Frequently | 8 (8%) | 0 (0%) | 6 (6%) | 3 (3%) | 5 (5%) | 3 (3%) | 5 (5%) | 3 (3%) | 4 (4%) | 4 (4%) |
| Always | 2 (2%) | 1 (1%) | 4 (4%) | 0 (0%) | 4 (4%) | 0 (0%) | 4 (4%) | 0 (0%) | 5 (5%) | 0 (0%) |
| Missing | 14 (13%) | 0 (0%) | 5 (5%) | 0 (0%) | 15 (14%) | 1 (1%) | 16 (15%) | 4 (4%) | 25 (24%) | 11 (11%) |
| **2) Unhappy about your sexual relationship(s)** | | | | | | | | | | |
| Never | 39 (38%) | 45 (44%) | 38 (37%) | 46 (45%) | 32 (31%) | 47 (46%) | 37 (36%) | 42 (41%) | 27 (26%) | 44 (43%) |
| Rarely | 22 (21%) | 21 (21%) | 33 (32%) | 28 (27%) | 29 (28%) | 27 (26%) | 24 (23%) | 34 (33%) | 20 (19%) | 22 (22%) |
| Occasionally | 24 (23%) | 26 (25%) | 23 (22%) | 19 (19%) | 18 (17%) | 20 (20%) | 18 (17%) | 12 (12%) | 22 (21%) | 17 (17%) |
| Frequently | 3 (3%) | 8 (8%) | 2 (2%) | 8 (8%) | 6 (6%) | 5 (5%) | 5 (5%) | 8 (8%) | 5 (5%) | 7 (7%) |
| Always | 2 (2%) | 2 (2%) | 3 (3%) | 1 (1%) | 4 (4%) | 2 (2%) | 4 (4%) | 2 (2%) | 5 (5%) | 1 (1%) |
| Missing | 14 (13%) | 0 (0%) | 5 (5%) | 0 (0%) | 15 (14%) | 1 (1%) | 16 (15%) | 4 (4%) | 25 (24%) | 11 (11%) |
| **3) Guilty about your sexual difficulties** | | | | | | | | | | |
| Never | 53 (51%) | 55 (54%) | 46 (44%) | 66 (65%) | 37 (36%) | 66 (65%) | 43 (41%) | 66 (65%) | 39 (38%) | 59 (58%) |
| Rarely | 10 (10%) | 28 (27%) | 19 (18%) | 19 (19%) | 22 (21%) | 20 (20%) | 15 (14%) | 14 (14%) | 15 (14%) | 15 (15%) |
| Occasionally | 11 (11%) | 13 (13%) | 16 (15%) | 13 (13%) | 16 (15%) | 6 (6%) | 17 (16%) | 13 (13%) | 10 (10%) | 12 (12%) |
| Frequently | 12 (12%) | 4 (4%) | 14 (13%) | 4 (4%) | 9 (9%) | 8 (8%) | 6 (6%) | 5 (5%) | 7 (7%) | 5 (5%) |
| Always | 4 (4%) | 2 (2%) | 4 (4%) | 0 (0%) | 5 (5%) | 1 (1%) | 7 (7%) | 0 (0%) | 8 (8%) | 0 (0%) |
| Missing | 14 (13%) | 0 (0%) | 5 (5%) | 0 (0%) | 15 (14%) | 1 (1%) | 16 (15%) | 4 (4%) | 25 (24%) | 11 (11%) |
| **4) Frustrated by your sexual problems** | | | | | | | | | | |
| Never | 54 (52%) | 54 (53%) | 49 (47%) | 63 (62%) | 38 (37%) | 69 (68%) | 42 (40%) | 70 (69%) | 35 (34%) | 62 (61%) |
| Rarely | 14 (13%) | 32 (31%) | 25 (24%) | 22 (22%) | 20 (19%) | 12 (12%) | 22 (21%) | 19 (19%) | 17 (16%) | 15 (15%) |
| Occasionally | 11 (11%) | 11 (11%) | 13 (12%) | 13 (13%) | 19 (18%) | 14 (14%) | 14 (13%) | 7 (7%) | 15 (14%) | 11 (11%) |
| Frequently | 9 (9%) | 4 (4%) | 7 (7%) | 3 (3%) | 6 (6%) | 6 (6%) | 3 (3%) | 2 (2%) | 6 (6%) | 2 (2%) |
| Always | 2 (2%) | 1 (1%) | 5 (5%) | 1 (1%) | 6 (6%) | 0 (0%) | 6 (6%) | 0 (0%) | 6 (6%) | 1 (1%) |
| Missing | 14 (13%) | 0 (0%) | 5 (5%) | 0 (0%) | 15 (14%) | 1 (1%) | 17 (16%) | 4 (4%) | 25 (24%) | 11 (11%) |
| **5) Stressed about sex** | | | | | | | | | | |
| Never | 52 (50%) | 61 (60%) | 48 (46%) | 65 (64%) | 35 (34%) | 62 (61%) | 44 (42%) | 67 (66%) | 40 (38%) | 59 (58%) |
| Rarely | 22 (21%) | 27 (26%) | 26 (25%) | 22 (22%) | 33 (32%) | 21 (21%) | 24 (23%) | 18 (18%) | 20 (19%) | 20 (20%) |
| Occasionally | 10 (10%) | 12 (12%) | 13 (12%) | 13 (13%) | 13 (12%) | 16 (16%) | 15 (14%) | 8 (8%) | 15 (14%) | 8 (8%) |
| Frequently | 5 (5%) | 2 (2%) | 11 (11%) | 2 (2%) | 5 (5%) | 2 (2%) | 2 (2%) | 4 (4%) | 2 (2%) | 4 (4%) |
| Always | 1 (1%) | 0 (0%) | 1 (1%) | 0 (0%) | 3 (3%) | 0 (0%) | 3 (3%) | 1 (1%) | 2 (2%) | 0 (0%) |
| Missing | 14 (13%) | 0 (0%) | 5 (5%) | 0 (0%) | 15 (14%) | 1 (1%) | 16 (15%) | 4 (4%) | 25 (24%) | 11 (11%) |
| **6) Inferior because of your sexual problems** | | | | | | | | | | |
| Never | 71 (68%) | 82 (80%) | 64 (62%) | 83 (81%) | 52 (50%) | 79 (77%) | 55 (53%) | 79 (77%) | 46 (44%) | 72 (71%) |
| Rarely | 8 (8%) | 15 (15%) | 21 (20%) | 13 (13%) | 24 (23%) | 13 (13%) | 18 (17%) | 15 (15%) | 18 (17%) | 14 (14%) |
| Occasionally | 5 (5%) | 3 (3%) | 9 (9%) | 4 (4%) | 5 (5%) | 8 (8%) | 8 (8%) | 2 (2%) | 10 (10%) | 3 (3%) |
| Frequently | 5 (5%) | 1 (1%) | 3 (3%) | 1 (1%) | 6 (6%) | 1 (1%) | 3 (3%) | 2 (2%) | 2 (2%) | 2 (2%) |
| Always | 0 (0%) | 1 (1%) | 2 (2%) | 1 (1%) | 1 (1%) | 0 (0%) | 4 (4%) | 0 (0%) | 3 (3%) | 0 (0%) |
| Missing | 15 (14%) | 0 (0%) | 5 (5%) | 0 (0%) | 16 (15%) | 1 (1%) | 16 (15%) | 4 (4%) | 25 (24%) | 11 (11%) |
| **7) Worried about sex** | | | | | | | | | | |
| Never | 52 (50%) | 62 (61%) | 45 (43%) | 63 (62%) | 44 (42%) | 64 (63%) | 47 (45%) | 64 (63%) | 46 (44%) | 59 (58%) |
| Rarely | 19 (18%) | 26 (25%) | 27 (26%) | 21 (21%) | 24 (23%) | 20 (20%) | 22 (21%) | 21 (21%) | 15 (14%) | 24 (24%) |
| Occasionally | 12 (12%) | 12 (12%) | 14 (13%) | 15 (15%) | 15 (14%) | 12 (12%) | 13 (12%) | 9 (9%) | 11 (11%) | 5 (5%) |
| Frequently | 5 (5%) | 2 (2%) | 9 (9%) | 3 (3%) | 3 (3%) | 5 (5%) | 2 (2%) | 4 (4%) | 4 (4%) | 2 (2%) |
| Always | 2 (2%) | 0 (0%) | 4 (4%) | 0 (0%) | 3 (3%) | 0 (0%) | 4 (4%) | 0 (0%) | 3 (3%) | 1 (1%) |
| Missing | 14 (13%) | 0 (0%) | 5 (5%) | 0 (0%) | 15 (14%) | 1 (1%) | 16 (15%) | 4 (4%) | 25 (24%) | 11 (11%) |
| **8) Sexually inadequate** | | | | | | | | | | |
| Never | 58 (56%) | 68 (67%) | 49 (47%) | 74 (73%) | 45 (43%) | 71 (70%) | 47 (45%) | 66 (65%) | 41 (39%) | 60 (59%) |
| Rarely | 14 (13%) | 22 (22%) | 28 (27%) | 15 (15%) | 22 (21%) | 15 (15%) | 20 (19%) | 23 (23%) | 19 (18%) | 20 (20%) |
| Occasionally | 12 (12%) | 8 (8%) | 14 (13%) | 9 (9%) | 12 (12%) | 12 (12%) | 12 (12%) | 8 (8%) | 13 (12%) | 8 (8%) |
| Frequently | 3 (3%) | 3 (3%) | 3 (3%) | 4 (4%) | 6 (6%) | 3 (3%) | 5 (5%) | 0 (0%) | 4 (4%) | 2 (2%) |
| Always | 3 (3%) | 1 (1%) | 5 (5%) | 0 (0%) | 4 (4%) | 0 (0%) | 4 (4%) | 1 (1%) | 2 (2%) | 1 (1%) |
| Missing | 14 (13%) | 0 (0%) | 5 (5%) | 0 (0%) | 15 (14%) | 1 (1%) | 16 (15%) | 4 (4%) | 25 (24%) | 11 (11%) |
| **9) Regretful about your sex life** | | | | | | | | | | |
| Never | 55 (53%) | 59 (58%) | 45 (43%) | 54 (53%) | 40 (38%) | 58 (57%) | 37 (36%) | 61 (60%) | 37 (36%) | 60 (59%) |
| Rarely | 17 (16%) | 18 (18%) | 28 (27%) | 20 (20%) | 23 (22%) | 24 (24%) | 20 (19%) | 19 (19%) | 17 (16%) | 16 (16%) |
| Occasionally | 13 (12%) | 17 (17%) | 12 (12%) | 22 (22%) | 16 (15%) | 9 (9%) | 18 (17%) | 10 (10%) | 15 (14%) | 10 (10%) |
| Frequently | 3 (3%) | 4 (4%) | 9 (9%) | 5 (5%) | 7 (7%) | 10 (10%) | 9 (9%) | 8 (8%) | 6 (6%) | 1 (1%) |
| Always | 2 (2%) | 4 (4%) | 5 (5%) | 1 (1%) | 3 (3%) | 0 (0%) | 4 (4%) | 0 (0%) | 4 (4%) | 4 (4%) |
| Missing | 14 (13%) | 0 (0%) | 5 (5%) | 0 (0%) | 15 (14%) | 1 (1%) | 16 (15%) | 4 (4%) | 25 (24%) | 11 (11%) |
| **10) Embarrassed about your sexual problems** | | | | | | | | | | |
| Never | 65 (62%) | 76 (75%) | 62 (60%) | 79 (77%) | 50 (48%) | 80 (78%) | 55 (53%) | 81 (79%) | 49 (47%) | 71 (70%) |
| Rarely | 11 (11%) | 20 (20%) | 24 (23%) | 16 (16%) | 21 (20%) | 13 (13%) | 19 (18%) | 13 (13%) | 17 (16%) | 12 (12%) |
| Occasionally | 6 (6%) | 4 (4%) | 7 (7%) | 5 (5%) | 13 (12%) | 2 (2%) | 8 (8%) | 3 (3%) | 8 (8%) | 6 (6%) |
| Frequently | 7 (7%) | 1 (1%) | 1 (1%) | 1 (1%) | 4 (4%) | 6 (6%) | 2 (2%) | 1 (1%) | 0 (0%) | 1 (1%) |
| Always | 1 (1%) | 1 (1%) | 5 (5%) | 1 (1%) | 1 (1%) | 0 (0%) | 4 (4%) | 0 (0%) | 5 (5%) | 1 (1%) |
| Missing | 14 (13%) | 0 (0%) | 5 (5%) | 0 (0%) | 15 (14%) | 1 (1%) | 16 (15%) | 4 (4%) | 25 (24%) | 11 (11%) |
| **11) Dissatisfied with your sex life** | | | | | | | | | | |
| Never | 43 (41%) | 41 (40%) | 36 (35%) | 43 (42%) | 34 (33%) | 46 (45%) | 36 (35%) | 41 (40%) | 27 (26%) | 48 (47%) |
| Rarely | 22 (21%) | 30 (29%) | 30 (29%) | 27 (26%) | 22 (21%) | 29 (28%) | 21 (20%) | 32 (31%) | 21 (20%) | 24 (24%) |
| Occasionally | 19 (18%) | 21 (21%) | 19 (18%) | 24 (24%) | 18 (17%) | 14 (14%) | 18 (17%) | 15 (15%) | 19 (18%) | 14 (14%) |
| Frequently | 4 (4%) | 7 (7%) | 9 (9%) | 6 (6%) | 10 (10%) | 10 (10%) | 7 (7%) | 10 (10%) | 7 (7%) | 2 (2%) |
| Always | 2 (2%) | 3 (3%) | 5 (5%) | 2 (2%) | 5 (5%) | 2 (2%) | 6 (6%) | 0 (0%) | 5 (5%) | 3 (3%) |
| Missing | 14 (13%) | 0 (0%) | 5 (5%) | 0 (0%) | 15 (14%) | 1 (1%) | 16 (15%) | 4 (4%) | 25 (24%) | 11 (11%) |
| **12) Angry about your sex life** | | | | | | | | | | |
| Never | 69 (66%) | 83 (81%) | 66 (63%) | 84 (82%) | 54 (52%) | 81 (79%) | 54 (52%) | 83 (81%) | 52 (50%) | 75 (74%) |
| Rarely | 14 (13%) | 13 (13%) | 16 (15%) | 14 (14%) | 22 (21%) | 12 (12%) | 23 (22%) | 8 (8%) | 17 (16%) | 9 (9%) |
| Occasionally | 3 (3%) | 3 (3%) | 10 (10%) | 1 (1%) | 8 (8%) | 6 (6%) | 6 (6%) | 4 (4%) | 4 (4%) | 5 (5%) |
| Frequently | 1 (1%) | 2 (2%) | 4 (4%) | 2 (2%) | 3 (3%) | 2 (2%) | 2 (2%) | 3 (3%) | 3 (3%) | 1 (1%) |
| Always | 2 (2%) | 1 (1%) | 3 (3%) | 1 (1%) | 2 (2%) | 0 (0%) | 3 (3%) | 0 (0%) | 3 (3%) | 1 (1%) |
| Missing | 15 (14%) | 0 (0%) | 5 (5%) | 0 (0%) | 15 (14%) | 1 (1%) | 16 (15%) | 4 (4%) | 25 (24%) | 11 (11%) |
| **13) Bothered by low sexual desire** | | | | | | | | | | |
| Never | 41 (39%) | 43 (42%) | 41 (39%) | 49 (48%) | 29 (28%) | 46 (45%) | 33 (32%) | 50 (49%) | 30 (29%) | 42 (41%) |
| Rarely | 17 (16%) | 21 (21%) | 21 (20%) | 22 (22%) | 19 (18%) | 25 (25%) | 17 (16%) | 21 (21%) | 15 (14%) | 25 (25%) |
| Occasionally | 19 (18%) | 28 (27%) | 13 (12%) | 25 (25%) | 23 (22%) | 23 (23%) | 21 (20%) | 18 (18%) | 20 (19%) | 17 (17%) |
| Frequently | 7 (7%) | 7 (7%) | 15 (14%) | 6 (6%) | 12 (12%) | 4 (4%) | 12 (12%) | 7 (7%) | 7 (7%) | 6 (6%) |
| Always | 6 (6%) | 3 (3%) | 9 (9%) | 0 (0%) | 6 (6%) | 3 (3%) | 5 (5%) | 2 (2%) | 7 (7%) | 1 (1%) |
| Missing | 14 (13%) | 0 (0%) | 5 (5%) | 0 (0%) | 15 (14%) | 1 (1%) | 16 (15%) | 4 (4%) | 25 (24%) | 11 (11%) |
| Data are presented as n/N (%) for categorical measures. RRSO= Risk-Reducing Salpingo-Oophorectomy. | | | | | | | | | | |
