# Supplementary material for: Sexual Function, Activity and Distress 24 Months After Surgical Menopause: What Happens After Menopause (WHAM)—A Prospective Controlled Study
Source: BJOG. 2026 Jan 22;133(6):1188–99. doi: 10.1111/1471-0528.70158 (PMC13040429; doi:10.1111/1471-0528.70158)
Supplement: Supplementary file 5 — Table S5: Baseline and 24‐month follow‐up sexual activity status in Female Sexual Function Index (FSFI). [file BJO-133-1188-s002.docx]

**S5. Baseline and 24-month follow-up sexual activity status in Female Sexual Function Index (FSFI).**

|  | Sexually active at 24 months | Sexually inactive at 24 months | Total |
| --- | --- | --- | --- |
| Sexually active at baseline | 114 | 19 | 133 |
| Sexually inactive at baseline | 10 | 23 | 33 |
| Total | 124 | 42 | 166 |

4 missing FSFI scores at baseline, 37 missing FSFI scores at 24 months, including 1 woman with missing FSFI scores at both baseline and 24 months.
