# Supplementary material for: Sexual Function, Activity and Distress 24 Months After Surgical Menopause: What Happens After Menopause (WHAM)—A Prospective Controlled Study
Source: BJOG. 2026 Jan 22;133(6):1188–99. doi: 10.1111/1471-0528.70158 (PMC13040429; doi:10.1111/1471-0528.70158)
Supplement: Supplementary file 6 — Table S6: Baseline and 24‐month follow‐up sexual activity status in Sexual Activity Questionnaire (SAQ). [file BJO-133-1188-s001.docx]

**S6. Baseline and 24-month follow-up sexual activity status in Sexual Activity Questionnaire (SAQ).**

|  | Sexually active at 24 months | Sexually inactive at 24 months | Total |
| --- | --- | --- | --- |
| Sexually active at baseline | 112 | 16 | 128 |
| Sexually inactive at baseline | 7 | 26 | 33 |
| Total | 119 | 42 | 161 |

14 missing SAQ scores at baseline, 35 missing SAQ scores at 24 months, including 4 women with missing SAQ scores at both baseline and 24 months.
