# Supplementary material for: Sexual Function, Activity and Distress 24 Months After Surgical Menopause: What Happens After Menopause (WHAM)—A Prospective Controlled Study
Source: BJOG. 2026 Jan 22;133(6):1188–99. doi: 10.1111/1471-0528.70158 (PMC13040429; doi:10.1111/1471-0528.70158)
Supplement: Supplementary file 7 — Table S7: Descriptive statistics of outcomes in sexually active participants by study group and timepoint. [file BJO-133-1188-s007.docx]

**S7. Descriptive statistics of outcomes in sexually active participants by study group and timepoint.**

| **Outcome** | **RRSO (N=104)** | | | | | **Comparison (N=102)** | | | | |
| --- | --- | --- | --- | --- | --- | --- | --- | --- | --- | --- |
|  | **Baseline** | **3 months** | **6 months** | **12 months** | **24 months** | **Baseline** | **3 months** | **6 months** | **12 months** | **24 months** |
| **FSFI ^a^** | **N=77** | **N=73** | **N=74** | **N=67** | **N=58** | **N=80** | **N=80** | **N=78** | **N=79** | **N=68** |
| **Primary outcome** | | | | | | | | | | |
| Sexual dysfunction | 14 (18%) | 27 (37%) | 31 (42%) | 25 (37%) | 24 (41%) | 20 (25%) | 18 (23%) | 16 (21%) | 20 (25%) | 19 (28%) |
| **Secondary outcomes** | | | | | | | | | | |
| Overall score | 30.0 (27.2-32.4) | 27.7 (23.7-31.4) | 28.1 (23.7-30.3) | 27.9 (24.7-31.5) | 27.6 (23.1-30.4) | 29.5 (26.5-31.8) | 30.3 (27.0-32.4) | 30.3 (27.6-31.8) | 30.3 (26.5-31.9) | 30.3 (25.2-32.4) |
| Desire score  (all participants) | 3.6 (2.4-4.2) | 3.0 (2.4-3.6) | 3.6 (2.4-3.6) | 3.0 (2.4-3.6) | 2.7 (1.8-3.6) | 3.6 (2.4-3.6) | 3.6 (2.4-3.6) | 3.0 (2.4-3.6) | 3.6 (2.4-3.6) | 3.0 (2.4-4.2) |
| Desire score ^ | 3.6 (2.4-4.2) | 3.0 (2.4-4.2) | 3.6 (2.4-3.6) | 3.6 (2.4-3.6) | 3.0 (2.4-3.6) | 3.6 (2.7-3.6) | 3.6 (3.0-4.2) | 3.6 (3.0-3.6) | 3.6 (3.0-3.6) | 3.6 (2.4-4.2) |
| Arousal score | 4.8 (4.2-5.7) | 4.5 (3.6-5.4) | 4.5 (3.6-5.1) | 4.5 (3.6-5.4) | 4.2 (3.3-5.1) | 4.8 (4.5-5.7) | 5.1 (4.5-5.7) | 5.4 (4.5-5.7) | 5.1 (4.5-5.7) | 5.1 (3.9-5.7) |
| Lubrication score | 6.0 (5.4-6.0) | 5.1 (3.9-6.0) | 5.1 (3.9-6.0) | 5.4 (4.2-6.0) | 5.4 (4.5-5.7) | 5.7 (5.3-6.0) | 5.7 (5.1-6.0) | 6.0 (5.4-6.0) | 5.7 (5.1-6.0) | 6.0 (5.0-6.0) |
| Orgasm score | 5.6 (4.8-6.0) | 5.2 (4.0-5.6) | 5.2 (4.0-5.6) | 5.2 (4.0-5.6) | 4.8 (3.6-6.0) | 5.6 (4.8-6.0) | 5.4 (4.6-6.0) | 5.6 (4.4-6.0) | 5.2 (4.8-6.0) | 5.6 (4.6-6.0) |
| Satisfaction score | 5.2 (4.8-6.0) | 5.2 (4.0-6.0) | 4.8 (4.0-6.0) | 5.2 (4.0-6.0) | 4.8 (3.2-5.6) | 4.8 (4.0-5.6) | 4.8 (4.0-5.6) | 4.8 (4.0-5.2) | 4.8 (4.0-5.6) | 5.2 (4.8-5.6) |
| Pain score | 6.0 (5.6-6.0) | 6.0 (4.8-6.0) | 6.0 (4.4-6.0) | 6.0 (5.2-6.0) | 6.0 (5.2-6.0) | 6.0 (5.2-6.0) | 6.0 (5.6-6.0) | 6.0 (6.0-6.0) | 6.0 (5.6-6.0) | 6.0 (5.6-6.0) |
| **SAQ ^b^** | **N=69** | **N=78** | **N=71** | **N=69** | **N=60** | **N=79** | **N=77** | **N=72** | **N=74** | **N=67** |
| Pleasure score | 11.0 (8.5-14.5) | 12.0 (9.0-16.0) | 12.0 (9.0-16.0) | 12.5 (10.0-16.0) | 13.5 (10.0-16.0) | 12.0 (9.0-14.0) | 11.0 (8.0-13.0) | 12.0 (8.5-15.0) | 12.0 (9.0-14.0) | 11.5 (9.0-15.0) |
| Discomfort score | 8.0 (7.0-8.0) | 7.0 (5.0-8.0) | 7.0 (5.0-8.0) | 7.0 (6.0-8.0) | 7.0 (5.0-8.0) | 7.0 (6.0-8.0) | 7.0 (7.0-8.0) | 8.0 (7.0-8.0) | 8.0 (7.0-8.0) | 8.0 (7.0-8.0) |
| Habit score | 3.0 (3.0-3.0) | 3.0 (3.0-4.0) | 3.0 (3.0-4.0) | 3.0 (3.0-4.0) | 3.0 (3.0-4.0) | 3.0 (3.0-3.0) | 3.0 (3.0-3.0) | 3.0 (3.0-3.0) | 3.0 (3.0-4.0) | 3.0 (3.0-4.0) |
| **FSDS-R ^c^** | **N=90** | **N=99** | **N=89** | **N=88** | **N=79** | **N=102** | **N=102** | **N=101** | **N=98** | **N=91** |
| Total score | 6.5 (1.0-15.0) | 9.0 (2.0-18.0) | 10.0 (2.0-19.0) | 9.5 (1.0-18.2) | 10.0 (1.0-20.0) | 7.0 (2.0-14.0) | 6.0 (1.0-13.0) | 4.0 (0.0-12.0) | 4.0 (1.0-12.0) | 4.0 (1.0-13.0) |
| Sexually related distress | 28 (31%) | 41 (41%) | 44 (49%) | 42 (48%) | 38 (48%) | 38 (37%) | 33 (32%) | 30 (30%) | 30 (31%) | 26 (29%) |
| Data are presented as median (IQR) for continuous measures, and n (%) for categorical measures. RRSO=Risk-reducing salpingo-oophowrectomy; IQR=interquartile range. FSFI= Female Sexual Function Index. SAQ= Sexual Activity Questionnaire. FSDS-R= Revised Female Sexual Distress Scale.  ^a^ Analyses were conducted among participants who were sexually active at each timepoint except for the desire domain where analysis was conducted among all participants with available data in the dataset. A participant is considered sexually active if they do not indicate either “no sexual activity” on any of the 15 FSFI items (12 items – Q7-18) or “did not attempt intercourse” (3 items – Q21-23) within the past 4 weeks.  ^b^ Analyses were conducted among participants who were sexually active at each timepoint. A participant is considered sexually active if they responded to being 'involved in sexual relationship' (i.e. patients who responded to the question 3 [Do you engage in sexual activity with anyone at the moment?] of Section 1 of the SAQ), or did not indicate 'not at all' to the question 9 [How often did you engage in sexual activity this month?] whenever Section 1 of the SAQ was administrated to participants.  ^c^ Analyses were conducted among participants who completed FSDS-R questionnaire at each timepoint.  ^ Sensitivity analysis for desire domain based on participants who were sexually active in FSFI at each timepoint | | | | | | | | | | |
