# Supplementary material for: Sexual Function, Activity and Distress 24 Months After Surgical Menopause: What Happens After Menopause (WHAM)—A Prospective Controlled Study
Source: BJOG. 2026 Jan 22;133(6):1188–99. doi: 10.1111/1471-0528.70158 (PMC13040429; doi:10.1111/1471-0528.70158)
Supplement: Supplementary file 8 — Table S8: Missing data patterns by timepoint. [file BJO-133-1188-s008.docx]

**S8. Missing data patterns by timepoint.**

|  | **Baseline, n(%)** | **3 months, n(%)** | **6 months , n(%)** | **12 months, n(%)** | **24 months, n(%)** |
| --- | --- | --- | --- | --- | --- |
| **Number of patients in the study** | 206 (100%) | 204 (99%) | 200 (97%) | 196 (95%) | 178 (86%) |
| Missing by design (not sexually active) | | | | | |
| FSFI | 45 (22%) | 45 (22%) | 39 (19%) | 39 (19%) | 43 (21%) |
| SAQ | 44 (21%) | 44 (21%) | 47 (23%) | 43 (21%) | 44 (21%) |
| Missing for other reasons ^a^ | | | | | |
| FSFI | 4 (2%) | 8 (4%) | 15 (7%) | 21 (10%) | 37 (18%) |
| SAQ | 14 (7%) | 7 (3%) | 16 (8%) | 20 (10%) | 35 (17%) |
| FSDS-R | 14 (7%) | 5 (2%) | 16 (8%) | 20 (10%) | 36 (17%) |
| Number of patients with analysable data | | | | | |
| FSFI | 157 (76%) | 153 (74%) | 152 (74%) | 146 (71%) | 126 (61%) |
| SAQ | 148 (72%) | 155 (75%) | 143 (69%) | 143 (69%) | 127 (62%) |
| FSDS-R | 192 (93%) | 201 (98%) | 190 (92%) | 186 (90%) | 170 (83%) |
| n=number of participants; FSFI=Female Sexual Function Index; SAQ=Sexual Activity Questionnaire; FSDS-R=Revised Female Sexual Distress Scale | | | | | |
| ^a^ Other reasons include incomplete questionnaire, insufficient for imputation, withdrawal. | | | | | |
